# ReFOLD3: refinement of 3D protein models with gradual restraints based on predicted local quality and residue contacts

**DOI:** 10.1093/nar/gkab300

**Published:** 2021-05-01

**Authors:** Recep Adiyaman, Liam J McGuffin

**Affiliations:** School of Biological Sciences, University of Reading, Whiteknights, Reading RG6 6AS, UK; School of Biological Sciences, University of Reading, Whiteknights, Reading RG6 6AS, UK

## Abstract

ReFOLD3 is unique in its application of gradual restraints, calculated from local model quality estimates and contact predictions, which are used to guide the refinement of theoretical 3D protein models towards the native structures. ReFOLD3 achieves improved performance by using an iterative refinement protocol to fix incorrect residue contacts and local errors, including unusual bonds and angles, which are identified in the submitted models by our leading ModFOLD8 model quality assessment method. Following refinement, the likely resulting improvements to the submitted models are recognized by ModFOLD8, which produces both global and local quality estimates. During the CASP14 prediction season (May–Aug 2020), we used the ReFOLD3 protocol to refine hundreds of 3D models, for both the refinement and the main tertiary structure prediction categories. Our group improved the global and local quality scores for numerous starting models in the refinement category, where we ranked in the top 10 according to the official assessment. The ReFOLD3 protocol was also used for the refinement of the SARS-CoV-2 targets as a part of the CASP Commons COVID-19 initiative, and we provided a significant number of the top 10 models. The ReFOLD3 web server is freely available at https://www.reading.ac.uk/bioinf/ReFOLD/.

## Graphical Abstract

**Graphical abstract:**
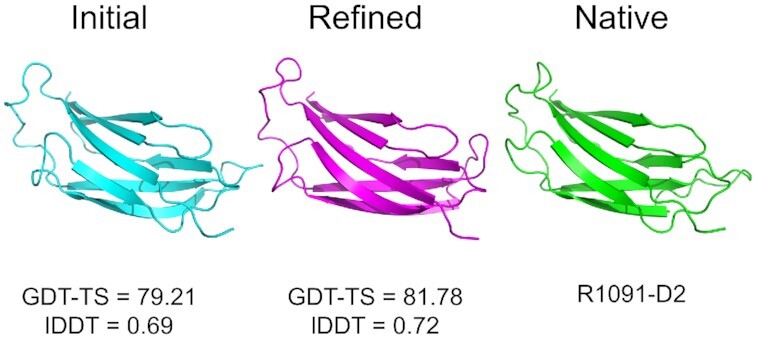
Refinement of a 3D model using ReFOLD3.

## INTRODUCTION

*In silico* modelling of protein structures provides a potential solution for bridging the protein sequence-structure gap. Although protein structures can now be predicted with high accuracy, using either advanced template based or deep learning-based methods, the resulting 3D models may still include significant local errors including unfavourable contacts, irregular hydrogen bonds, geometrical clashes and unusual angles. These errors may limit the usage of the modelled structures in cases where high accuracy is required, such as in drug discovery and protein engineering (1–3). The refinement of predicted protein structures refers to the correction of the local errors and improvement of the overall quality of theoretical 3D models, which is often considered as the ‘last mile’ for structure prediction (4).

Methods for the refinement of 3D protein models aim to increase model accuracy by fixing the local errors with the adjustment of secondary structure and modification of the sidechain interactions (3,5). Ironically, until recent years, the refinement of 3D models has more often than not resulted in decreases in average model accuracy and it remains a challenge for refinement groups to consistently improve all 3D models across all categories of target difficulty (3,6).

The employment of physics-based molecular dynamics (MD) simulations has been the main stay for the non-server-based refinement methods. Since the CASP9 experiment, leading MD methods have generated refined 3D models by taking advantage of new force fields, parallel computing and restraint strategies (3). Although the MD-based protocols have been used by many top-performing groups in recent CASP experiments, they have suffered from a high computational time cost and the inadequacy of force fields for directing the generation of the 3D models towards the native basin (2–4,7,8). Both the automated server-based and non-server-based approaches for refinement sample dozens of 3D models in many alternative conformations (9). The most native like conformation among all 3D models are subsequently identified in the scoring stage (3). However, it is often problematic to recognise the improved models using either energy functions or model quality assessment programs (MQAPs), as the similarity among the 3D models generated by the sampling approaches (3).

The original ReFOLD server (10) was developed by our group to refine 3D models using a hybrid approach, which included both rapid and MD-based sampling and leading model quality estimates, to produce the predicted local and global quality scores for each sampled 3D model. The first protocol included the refinement of the initial structure using i3Drefine in 20 refinement cycles (1,11). The second protocol accommodated an MD-based protocol inspired by that of Feig and Mirjalili (7,12,13), but using more modest computational resources compared to the supercomputer scale resources, which were originally used. All 3D models generated by these sampling approaches were ranked using ModFOLD6 (14) at the last stage. The original ReFOLD method (10) was used for the refinement of CASP12 targets, and it showed promising performance, boosting the accuracy of our prediction pipeline (10). Nevertheless, structural drifts from the native state often occurred, especially for the templated based modelling (TBM) targets (10).

The ModFOLD server (14–17) has been a leading approach for predicting the local and global quality of theoretical 3D models for more than a decade, according to both the CASP and CAMEO experiments (2,14–17). The local quality estimates provide significant information about the predicted accuracy score for each residue in each 3D model. In CASP13, ReFOLD2 was developed, which utilized a fixed restraint threshold, based on the per-residue accuracy scores produced by ModFOLD7 (the predicted distances from the native structure for each C-alpha atom), in order to guide the MD-based protocol. The CASP13 targets were relatively larger compared to the previous CASP experiments (3,10,18), and we observed that fixed restraint thresholds were not always appropriate for larger targets containing domains with varying accuracy. Therefore, with ReFOLD3, we developed a more targeted, novel gradual restraint strategy based on the local quality estimates for each starting model, which considered the required level of refinement for each individual residue.

In CASP13, contact prediction methods showed significant progress and reached up to 70% accuracy following the application of advanced deep learning methods (19–21). Residue–residue contact prediction methods have been used for the prediction of 3D models, protein–ligand interactions (22) and 3D model quality estimation (23–26), and more recently, the performance of the tertiary structure prediction has been boosted by contact prediction methods (19–21). Thus for ReFOLD3, we also utilized contact predictions for the refinement of the predicted 3D models with the application of an additional gradual restraint strategy based on our contact distance agreement (CDA) score (14). The CDA score measures the agreement between the contacts, which were predicted by the DeepMetaPSICOV method (21,27,28), and the contacts between residues in the predicted 3D model, measured according to their Euclidean distance (14). The CDA score has been a local scoring component of the ModFOLD server since CASP11, and it will become increasingly important as contact prediction accuracy improves (14,21,27–29).

In CASP14, ReFOLD3 gave us a performance boost in the main tertiary structure prediction category, where it enabled us to further improve the quality of some of the very best initial server models. Our group ranked in the top 10 in the refinement category itself, according to the official assessment. This was significant in the context of the very high-quality models of tertiary structures produced by many modelling groups. In CASP14, the models were much harder to refine as there was less room for improvement. Despite this major progress in modelling, most server models still contained significant local errors, which were both detectable by our ModFOLD8 method and fixable using ReFOLD3. It is notable that ReFOLD3 performed better at refining the most highly accurate starting models and it ranked within the top 5 refinement approaches, for models with GDT-TS scores >70. As 3D models of proteins become more widely used, it is important that biologists have access to freely available tools, such as ReFOLD3, in order to confidently identify and fix local errors in their theoretical models and bring them even closer to experimental quality.

## MATERIALS AND METHODS

ReFOLD3, consisted of four protocols, which included improvements on the original version (10). The major improvement for ReFOLD3 was the accommodation of the two new comprehensive MD-based strategies (Supplementary Figure S1). The first and final protocol used i3Drefine (1,11,30) to refine the input model with 20 rapid iterations, the second and third protocols both employed more CPU/GPU intensive molecular dynamic simulation strategies, and models were ranked and selected at each stage using our ModFOLD8 server (14,16).

The second protocol included the introduction of molecular dynamics simulations that were guided by the per-residue accuracy scores obtained from ModFOLD8 (14,16,31). For the gradual restraint strategy, it was assumed that the regions identified as highly accurate should be restrained by applying a stronger harmonic positional restraint to prevent deviations from the native basin (3,7,13). A weaker restraint was also applied to the poorly predicted regions to allow for increases in the quality of these regions towards the native state (Supplementary Figure S2A). Thus, the gradual restraints ranged from weak (0.05 kCal/mol/Å^2^) to strong (1 kCal/mol/Å^2^) harmonic positional restraints, which were applied to all atoms, including C-alphas, according to the distribution of the per-residue accuracy scores produced by ModFOLD8 (Supplementary Table S1) (3,7,10,18,31).

For the third protocol, a similar gradual restraint strategy was used to guide the MD simulation, but this time it was based on the residue-residue contact predictions. We utilized the CDA score method (14,31), which is based on the agreement between the residue contacts predicted by DeepMetaPSICOV (21) and the contacts in the model. If the CDA score was high, a stronger restraint was applied to preserve those residue contacts in the predicted 3D model. Lower CDA scores indicated where residues were likely to be further away from the native structure, and so weaker restraints were applied (Supplementary Table S2 and Supplementary Figure S2B).

The MD-based protocols were carried out at normal cellular conditions at 298 K with 1 bar of pressure in explicit solvent using nanoscale molecular dynamics (NAMD) (32) version 2.10 in Graphics processing unit (GPU) mode. The CHARMM22/27 force field (33) and the TIP3P water model (34) were also used for the simulation of the protein system. The system was also neutralized by inserting Na+ or Cl- ions to balance the net charge using Particle Mesh Ewald (PME) (35). The non-bonded interactions (mostly van der Waal’s) were cut off by 12 Å to the exclusion of bonded interactions by using the CHARMM27 default parameter file with the switching distance of 10 Å (10). Using the pairlistdist function with 14 Å distance between atom pairs for inclusion in pair lists made the switching function more efficient (10). The rigidBonds functions were also used to rigidify hydrogen bonds with a 2 fs timestep (10). The system’s electrostatics and the temperature were calculated by PME with the temperature control using Langevin dynamics under the NTP conditions (constant number of particles, temperature and pressure) (10,36). The correction of clashes and the minimization of the system were carried out by 1000 steps in the first step of the MD-based protocol (10). The minimization step was followed by the implementation of the defined MD simulation to refine each target (10). Four parallel simulations were run for 2 ns, making 8 ns in total for a target as in the original MD-based protocol of ReFOLD (10). After the completion of the simulation run, 164 refined models were generated per target by taking a snapshot every 50ps, for each MD-based protocol (10).

Refined models generated from the MD-based protocols were then assessed and ranked using ModFOLD8_rank (10,14,31). The fourth protocol was a combination of the MD-based approaches, where the top-ranked model from the second and third protocol was then further refined using i3Drefine (1,10,11,14,31). Finally, all of the refined models generated by each of these protocols and the input model were pooled and re-ranked again according to the ModFOLD8_rank global scores (1,10,11,14,31).

## RESULTS AND DISCUSSION

### Server inputs and outputs

**Figure 1.**
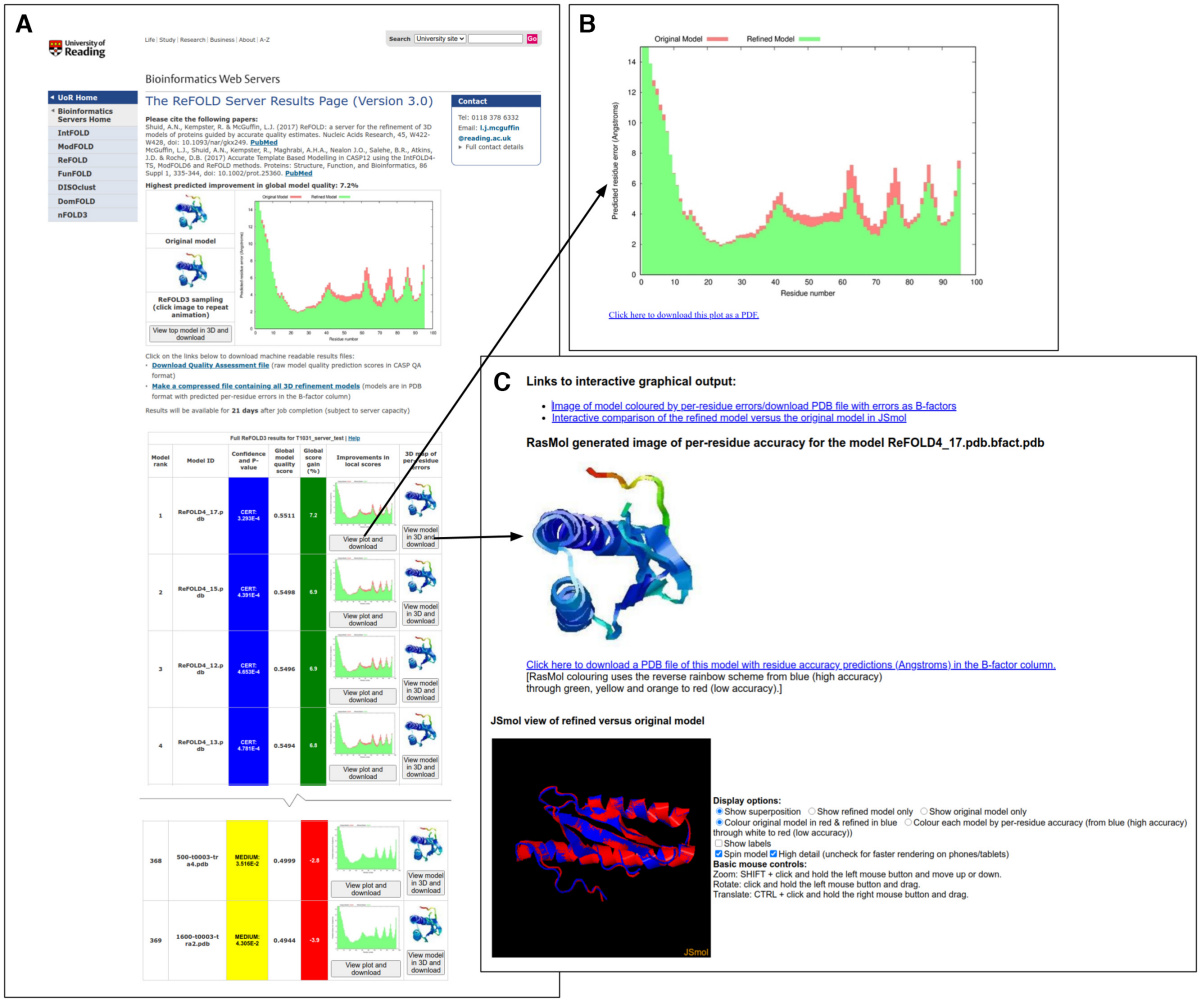
The ReFOLD3 server results page for an FM CASP14 target (T1031). In CASP14, the ReFOLD3 protocol was used to refine the top selected server models. (**A**) The results page provides the superposition of the top model and the input 3D model, a summary of the predicted scores and improvements in model quality estimation, along with the confidence intervals and *P*-value for each generated 3D model. After clicking on images or plots, users can download and view the data in detail (**B**) The plot of the predicted per-residue accuracy scores produced by ModFOLD8 for the top refined 3D model (green bars) compared with the original 3D model (red bars). Plots can also be downloaded as PDFs. (**C**) Interactive superposition of the top 3D model and the original 3D model, which are displayed in 3D using JSmol. Models can also be downloaded which including the per-residue error data in the B-factor column.

The required inputs for ReFOLD3 are the protein sequence and a 3D model (in PDB format) for refinement. Optionally, users may provide a name for their protein sequence and their email address. The ReFOLD3 server results page (Figure 1) includes an accurate estimate of the likely percentage improvement in model quality score for each refined 3D model, and it is unique in providing a series of individual per-residue error plots (Figure 1B), which show the local quality estimates for the refined models compared to the uploaded protein structure. Therefore, users may easily visualize the specific local improvements in every refined 3D model at a glance. Users may also click buttons to compare the refined and original 3D models interactively directly within the browser. The superposition of the refined models with the input models can be visualized using the JSmol/HTML5 framework. Therefore, models can be viewed in 3D directly within in the browser, including on mobile devices, without the requirement of any plugins (Figure 1C). The ReFOLD3 server will typically provide the results within ∼21 h for a target with 100 residues, with the MD simulation stages lasting ∼4 h (with Intel Xeon Platinum 8268 CPUs and Nvidia Tesla T4 GPUs). The server should complete the majority of refinement jobs within 48 h, once they are running.

### Independent benchmarking and cross validation

**Figure 2.**
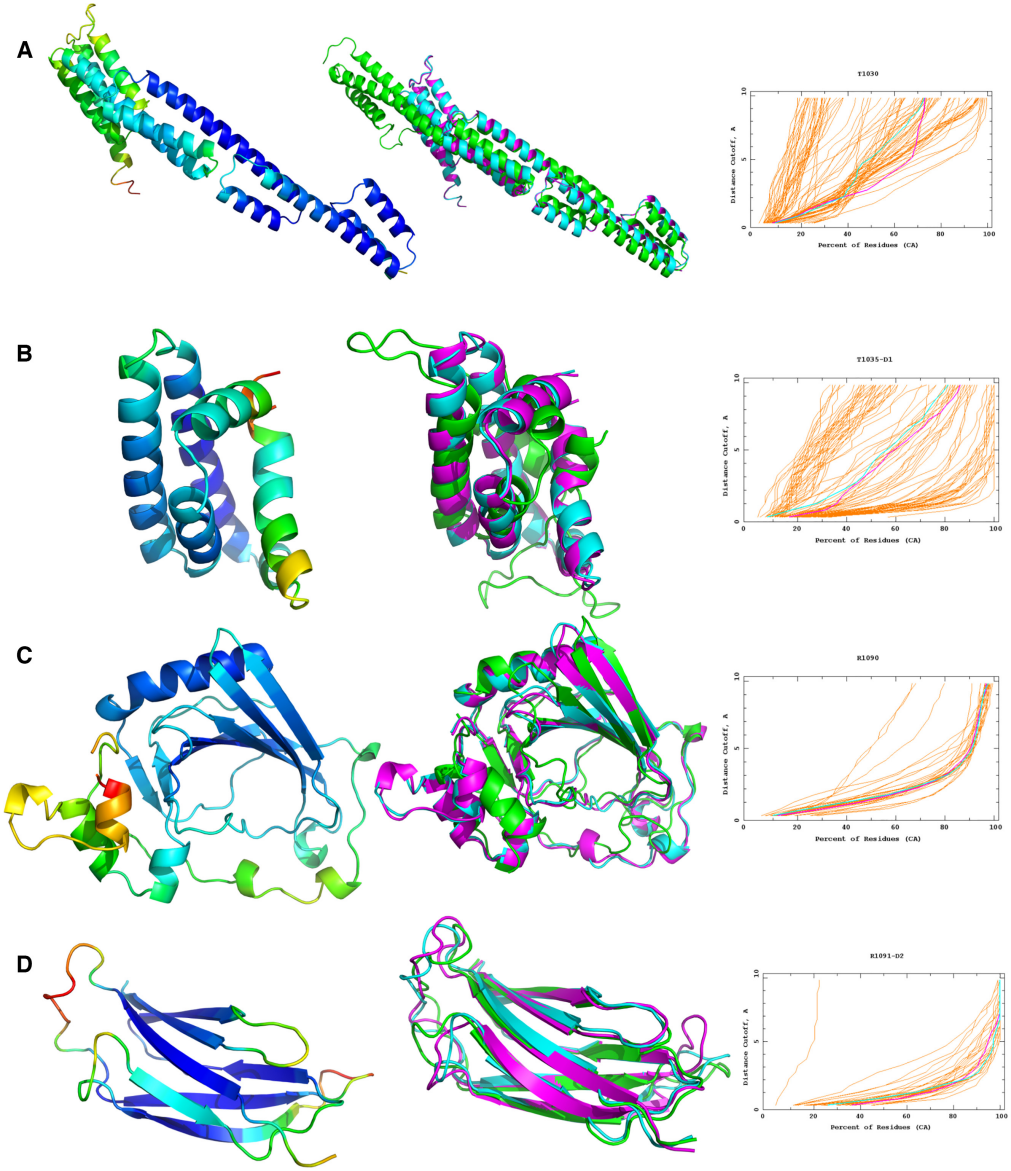
The refinement of four CASP14 targets using the ReFOLD3 protocol. In the left panels, the initial structures were coloured by the per-residue accuracy score produced by ModFOLD8. In the middle panels, superposition of the best predicted 3D server model selected by ModFOLD8 or the starting model provided by CASP in the refinement category (cyan), the top 3D model generated by ReFOLD3 (magenta) and native structure (green). In the right panels, GDT_TS plots for the comparison of the original 3D model (cyan) with the top 3D model generated by ReFOLD3 (magenta). (**A**) CASP14 regular TBM-hard target T1030: RaptorX_TS1 versus McGuffin_TS1, a GDT_TS improvement from 39.84 to 43.77. (**B**) CASP14 regular FM/TBM target T1035 domain 1: tFOLD-lDT_TS3 versus McGuffin_TS1, a GDT_TS improvement from 48.28 to 50.00. (**C**) CASP14 refinement FM target R10909: starting model versus McGuffin_TS1, a GDT_TS improvement from 65.61 to 67.06 (D) CASP14 refinement TBM-easy target R1091-D1: starting model versus McGuffin TS1, a GDT_TS improvement from 79.21 to 81.78. Images are created using PyMOL (http://www.pymol.org). GDT_TS plots are from https://www.predictioncenter.org/casp14/.

*CASP14 and CASP Commons 2020:* The ReFOLD3 protocol was used to provide a significant proportion of the top ten identified 3D models for the ten SARS-2-CoV targets in CASP Commons COVID-19 initiative (Supplementary Tables S3–S10) (1,10,11,14,31). ReFOLD3 was also subsequently blind tested by independent assessors in the CASP14 experiment (May–Aug 2020) (2,10,14,18,31). Our refinement pipeline was employed to refine the best server 3D models for each target, which were selected by ModFOLD8 in the regular prediction category, as well as the starting models provided by the CASP assessors for the refinement category itself (1,10,11,14,31). ReFOLD3 generated numerous improved models compared to the initial structures, and this enhanced the performance of our prediction pipeline in the CASP14 experiment (Table 1 and Figure 2) (1,10,11,14,31). ReFOLD3 was ranked as the ninth best approach according to the assessors’ formula (Supplementary Table S11) and tenth, according to the cumulative GDT-TS score in the CASP14 refinement category (Supplementary Table S12).

**Table 1.** The performance of the McGuffin group ReFOLD3 refinement pipeline for all regular CASP14 targets according to the GDT-TS and lDDT scores versus the starting model. The top ModFOLD8 selected server models were taken as the starting models that were refined using ReFOLD3 and submitted during the CASP14 experiment. Data are from https://www.predictioncenter.org/casp14/

CASP model ID				GDT-TS			LDDT		
Target ID	Prediction category	Starting model	Submitted model	Starting model	Refined model	Diff	Starting model	Refined model	Diff
T1024	TBM-easy	T1024TS326_2	T1024TS220_1	60.68	60.93	0.25	0.67	0.68	0.01
T1026	TBM-hard	T1026TS487_4-D1	T1026TS220_1-D1	68.49	68.15	-0.34	0.54	0.58	0.04
T1027	FM	T1027TS487_1-D1	T1027TS220_1-D1	36.87	37.12	0.25	0.4	0.4	0
T1029	FM	T1029TS487_1-D1	T1029TS220_1-D1	40.8	40.8	0	0.47	0.47	0
T1030	TBM-hard	T1030TS487_1	T1030TS220_1	39.84	43.77	3.93	0.6	0.62	0.02
T1031	FM	T1031TS209_5-D1	T1031TS220_1-D1	24.47	24.74	0.27	0.32	0.32	0
T1033	FM	T1033TS209_5-D1	T1033TS220_1-D1	46.25	45.25	-1	0.51	0.5	-0.01
T1034	TBM-easy	T1034TS487_1-D1	T1034TS220_1-D1	82.37	82.53	0.16	0.7	0.73	0.03
T1035	FM/TBM	T1035TS351_3-D1	T1035TS220_1-D1	48.28	50	1.72	0.5	0.5	0
T1037	FM	T1037TS337_1-D1	T1037TS220_1-D1	51.3	51.3	0	0.52	0.53	0.01
T1038	FM/TBM	T1038TS487_4	T1038TS220_1	26.45	26.58	0.13	0.37	0.36	-0.01
T1039	FM	T1039TS487_4-D1	T1039TS220_1-D1	34.63	34.78	0.15	0.35	0.38	0.03
T1040	FM	T1040TS351_3-D1	T1040TS220_1-D1	24.42	23.65	-0.77	0.38	0.38	0
T1041	FM	T1041TS377_3-D1	T1041TS220_1-D1	52.58	53	0.42	0.53	0.53	0
T1042	FM	T1042TS226_2-D1	T1042TS220_1-D1	54.98	54.62	-0.36	0.55	0.53	-0.02
T1043	FM	T1043TS487_3-D1	T1043TS220_1-D1	17.06	17.23	0.17	0.22	0.24	0.02
T1045s2	TBM-easy	T1045s2TS487_4-D1	T1045s2TS220_1-D1	69.28	70.03	0.75	0.63	0.65	0.02
T1046s1	FM/TBM	T1046s1TS487_3-D1	T1046s1TS220_1-D1	74.31	75	0.69	0.6	0.6	0
T1046s2	TBM-hard	T1046s2TS487_4-D1	T1046s2TS220_1-D1	75.53	76.06	0.53	0.57	0.63	0.06
T1047s1	FM	T1047s1TS075_1-D1	T1047s1TS220_1-D1	32.58	32.94	0.36	0.58	0.56	-0.02
T1047s2	FM/TBM	T1047s2TS487_5	T1047s2TS220_1	34.05	34.21	0.16	0.62	0.66	0.04
T1049	FM	T1049TS326_3-D1	T1049TS220_1-D1	63.25	63.43	0.18	0.57	0.58	0.01
T1050	TBM-easy	T1050TS487_1	T1050TS220_1	55.85	55.88	0.03	0.63	0.67	0.04
T1052	FM/TBM	T1052TS487_4	T1052TS220_1	54.48	54.15	-0.33	0.67	0.66	-0.01
T1053	FM/TBM	T1053TS238_4	T1053TS220_1	39.95	39.18	-0.77	0.54	0.53	-0.01
T1054	TBM-hard	T1054TS326_4-D1	T1054TS220_1-D1	67.48	68.71	1.23	0.66	0.68	0.02
T1055	FM/TBM	T1055TS238_5-D1	T1055TS220_1-D1	70.9	70.29	-0.61	0.57	0.55	-0.02
T1056	TBM-hard	T1056TS209_3-D1	T1056TS220_1-D1	54.44	53.25	-1.19	0.48	0.46	-0.02
T1057	TBM-easy	T1057TS351_5-D1	T1057TS220_1-D1	79.57	79.47	-0.1	0.68	0.68	0
T1060s3	TBM-hard	T1060s3TS487_4-D1	T1060s3TS220_1-D1	67.89	67.28	-0.61	0.61	0.64	0.03
T1061	FM/TBM	T1061TS326_4	T1061TS220_1	30.34	30.5	0.16	0.45	0.47	0.02
T1064	FM	T1064TS140_1-D1	T1064TS220_1-D1	20.65	20.65	0	0.22	0.23	0.01
T1065s1	TBM-hard	T1065s1TS487_1-D1	T1065s1TS220_1-D1	88.44	88.87	0.43	0.75	0.8	0.05
T1065s2	TBM-hard	T1065s2TS277_4-D1	T1065s2TS220_1-D1	90.31	91.07	0.76	0.78	0.78	0
T1067	TBM-hard	T1067TS351_3-D1	T1067TS220_1-D1	52.83	52.49	-0.34	0.51	0.49	-0.02
T1068	TBM-hard	T1068TS183_2-D1	T1068TS220_1-D1	57.26	55.17	-2.09	0.53	0.51	-0.02
T1073	TBM-easy	T1073TS140_5-D1	T1073TS220_1-D1	83.47	83.47	0	0.73	0.73	0
T1074	FM	T1074TS487_5-D1	T1074TS220_1-D1	35.8	35.98	0.18	0.38	0.39	0.01
T1076	TBM-easy	T1076TS487_4-D1	T1076TS220_1-D1	87.41	87.41	0	0.72	0.78	0.06
T1078	TBM-easy	T1078TS351_3-D1	T1078TS220_1-D1	76.74	76.55	-0.19	0.7	0.69	-0.01
T1079	TBM-easy	T1079TS487_2-D1	T1079TS220_1-D1	62.03	62.36	0.33	0.64	0.68	0.04
T1080	FM/TBM	T1080TS183_1-D1	T1080TS220_1-D1	24.81	25.19	0.38	0.41	0.41	0
T1082	FM/TBM	T1082TS487_5-D1	T1082TS220_1-D1	58.67	59.33	0.66	0.43	0.42	-0.01
T1083	TBM-hard	T1083TS364_4-D1	T1083TS220_1-D1	83.7	83.15	-0.55	0.71	0.71	0
T1084	TBM-hard	T1084TS252_2-D1	T1084TS220_1-D1	88.73	88.03	-0.7	0.76	0.75	-0.01
T1085	FM/TBM	T1085TS183_3	T1085TS220_1	35.97	35.78	-0.19	0.61	0.61	0
T1086	FM/TBM	T1086TS238_1	T1086TS220_1	41.8	42.06	0.26	0.73	0.71	-0.02
T1089	TBM-easy	T1089TS238_4-D1	T1089TS220_1-D1	65.45	65.92	0.47	0.59	0.59	0
T1090	FM	T1090TS487_1-D1	T1090TS220_1-D1	53.17	53.17	0	0.51	0.53	0.02
T1091	TBM-easy	T1091TS351_4	T1091TS220_1	26.62	26.67	0.05	0.62	0.62	0
T1092	TBM-easy	T1092TS319_3	T1092TS220_1	23.24	23.47	0.23	0.45	0.49	0.04
T1093	FM	T1093TS487_3	T1093TS220_1	25.44	25.4	-0.04	0.36	0.39	0.03
T1095	TBM-hard	T1095TS487_2-D1	T1095TS220_1-D1	41.86	42.09	0.23	0.56	0.61	0.05
T1096	FM	T1096TS252_1	T1096TS220_1	26.14	26.52	0.38	0.52	0.56	0.04
T1099	TBM-hard	T1099TS487_4-D1	T1099TS220_1-D1	57.3	55.76	-1.54	0.59	0.53	-0.06
T1101	TBM-easy	T1101TS487_4	T1101TS220_1	56.65	57.16	0.51	0.65	0.69	0.04
			Total	2943.86	2948.55	4.69	30.95	31.47	0.52

For the regular CASP14 prediction category, in our prediction pipeline, the top 3D model was selected by ModFOLD8 and then refined by the ReFOLD3 protocol in an attempt to further increase the accuracy of the predicted structure (1,10,11,14,31). From the results in Table 1, it is clear that ReFOLD3 managed to improve the quality for 68% of the top server 3D models selected by ModFOLD8, according to GDT-TS and lDDT scores (∑ΔGDT TS = 4.69, ∑ΔlDDT = 0.52). The GDT-TS score (37) is based on the superposition of C-alpha atoms between the predicted and native structure, while the lDDT score (38) calculates scores based on the atom–atom distance between the predicted or refined 3D model and the observed structure, considering all atoms (38). Thus, the results show that ReFOLD3 increased the accuracy of both the backbone and local regions for 68% of the structures. Furthermore, whereas the original version of ReFOLD was not successful in increasing the accuracy of the models for TBM targets in CASP12 (10), ReFOLD3 performed well across all target definitions in CASP14, including TBM (Figure 2, Supplementary Tables S13–S15).

The refinement of the best server 3D model in the regular prediction category is arguably closer to a real-world scenario, compared to refinement of the starting models provided by assessors in the refinement category. The starting models chosen by the CASP assessors for the refinement category, may have already been pre-refined in their modelling pipelines and they often represent the very highest quality starting models, which makes their further refinement much more difficult. Nevertheless, ReFOLD3 managed to improve the quality for 38% of the starting models in the refinement category according to the observed scores (Supplementary Table S16). Using ReFOLD3, our group ranked ninth in the refinement category for the regular time-frame targets, according to the official assessors’ formula (Supplementary Table S11). The ReFOLD3 method has also shown similar performance for each of the CASP14 target definitions, for the refinement category as well as the regular prediction category (Supplementary Table S17-S19). The datasets used for benchmarking are freely available for all to download via https://predictioncenter.org/download_area/CASP14/.

The performance of the ReFOLD3 was also analysed according to the size (number of amino acids) and the GDT-TS score intervals of the starting models by the CASP assessors in the refinement category (Supplementary Tables S20–S25). It is clear that the CASP14 refinement targets were much harder to refine overall and there was less room for improvement, as high-quality models of tertiary structures were produced by many modelling groups, which were then chosen by the assessors (20,39). However, it is important to note that many models still contained significant local errors, which were both detected and fixed using our ReFOLD3 pipeline (10,14,31). In that context, while many groups did not perform particularly well for targets with GDT-TS scores >70 (Supplementary Table S25), ReFOLD3 performed relatively better at refining the most highly accurate starting models and was ranked within the top 5 refinement approaches for targets with GDT-TS scores >60, according to the cumulative *Z*-score (Supplementary Tables S24 and S25).

## CONCLUSIONS

ReFOLD3 is one of the leading freely available fully automated servers for the refinement of theoretical 3D models of proteins. The method is unique in utilizing a gradual restraint strategy, based on both contact predictions and local quality estimation, to guide the refinement of protein models. Our application of gradual, rather than fixed, restraints has proved to be more successful for guiding our MD-simulations. As well as existing as an independent server, the ReFOLD3 method is integrated with the latest ModFOLD8 server (14,31). This integration enables users to identify the local errors in their 3D models and then correct them more conveniently, through the targeted improvement of specific regions. Using the ReFOLD3 server, non-expert users can easily visualize the likely improvements to their models, via intuitive per-residue error plots and 3D model superpositions.

## DATA AVAILABILITY

The ReFOLD3 server is freely available at: https://www.reading.ac.uk/bioinf/ReFOLD/

## Supplementary Material

gkab300_Supplemental_File
