# Supplementary material for: ReFOLD3: refinement of 3D protein models with gradual restraints based on predicted local quality and residue contacts
Source: Nucleic Acids Res. 2021 May 1;49(W1):W589–96. doi: 10.1093/nar/gkab300 (PMC8218204; doi:10.1093/nar/gkab300)
Supplement: gkab300_Supplemental_File [file gkab300_supplemental_file.docx]

Supplementary Figure S1. Flowchart of the ReFOLD3 refinement pipeline: the refinement of the starting model using i3Drefine (Protocol 1), the application of gradual restraint strategy based on the local quality estimation during the MD-based simulation (Protocol 2), the application of contact-assisted MD-based protocol during the MD simulation (Protocol 3), and the second round of i3Drefine iterative refinement (Protocol 4). All refined models were ranked by the ModFOLD8 server using the ModFOLD8_rank option (optimised for selecting the best top model), the refinement data are then compared with those of the original starting model and the results are displayed to the user via the web interface (Figure 1).


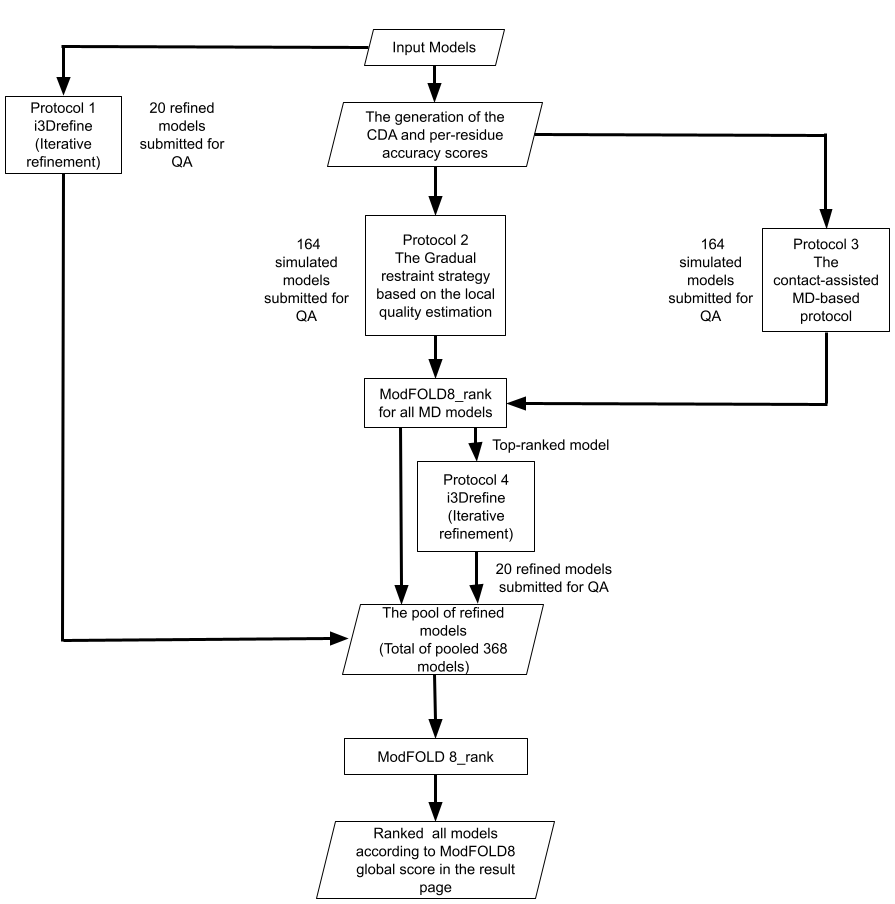


Supplementary Figure S2 The application of the gradual restraint strategies for the MD simulation based on the CDA score and per-residue accuracy score produced by ModFOLD8 for an example FM CASP14 target (R1049). (A) The application of the gradual restraint strategy based on the CDA score. 1. The starting model for the CASP14 target R1049 coloured by the CDA scores. 2. The starting model coloured by the occupancy column, where red and green regions applied strong restraints and blue and light blue regions applied weaker restraints depending on the CDA score. (B) The application of the gradual restraint strategy based on the per-residue accuracy score. 1. The starting model for the CASP14 target R1049 coloured by the per-residue accuracy score produced by ModFOLD8. 2. The starting model coloured by the occupancy column, where red and light green regions applied strong restraints and blue and light blue regions applied weaker restraints depending on the per-residue accuracy score.


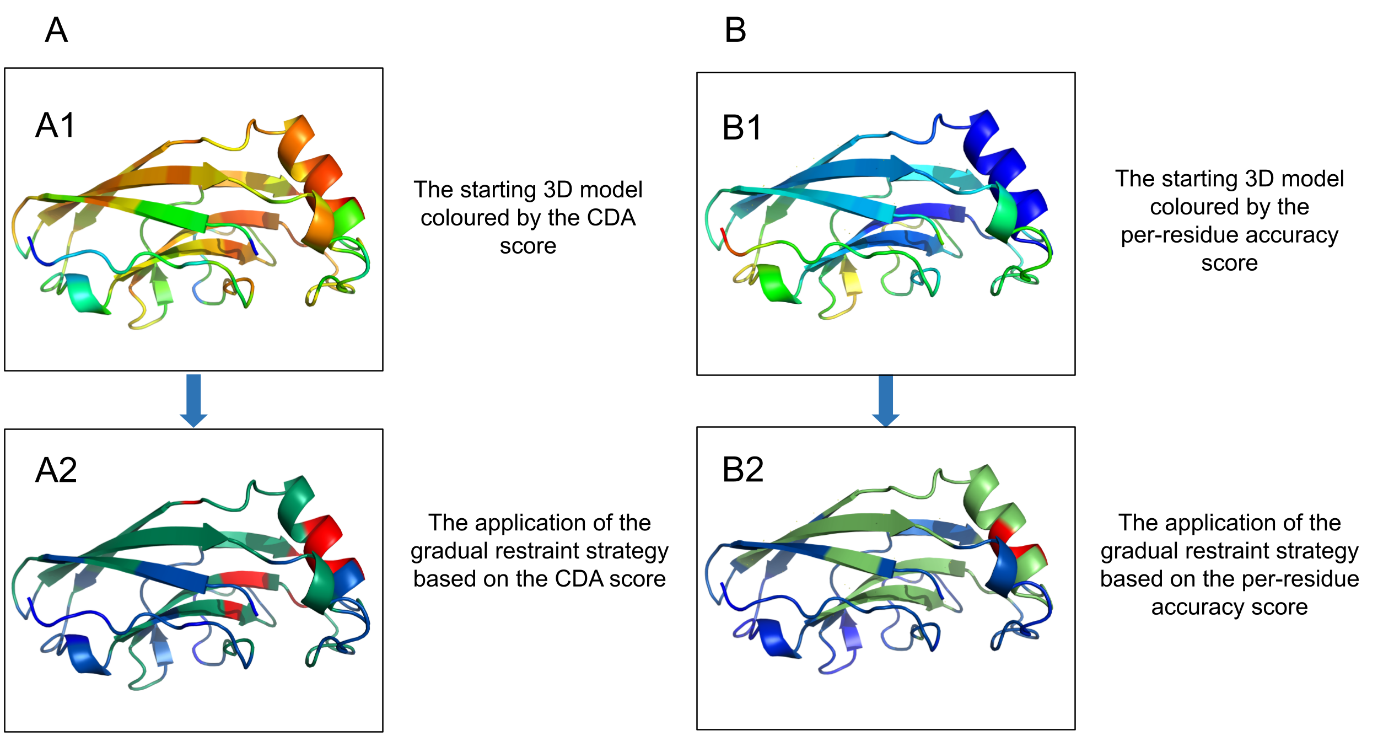


Supplementary Table S1. The application of the gradual restraint strategy based on the per-residue accuracy score produced by ModFOLD8

| The per-residue error (Å) | The force constant (kcal/mol/Å^2^ |
| --- | --- |
| 0-2 | 1 |
| 2-4 | 0.5 |
| 4-6 | 0.1 |
| 6-8 | 0.05 |
| 8 and above | 0 |

Supplementary Table S2. The application of the gradual restraint strategy based on the CDA score produced by ModFOLD8

| The CDA score | The force constant (kcal/mol/Å^2^) |
| --- | --- |
| 0.9-1 | 1 |
| 0.7-0.9 | 0.5 |
| 0.5-0.7 | 0.1 |
| 0.3-0.5 | 0.05 |
| 0-0.3 | 0 |

Supplementary Table S3. Official CASP Commons results for C1901 according to the consensus GDT-TS and LDDT scores for the top 20 models. Our group was registered as “McGuffin” in CASP Commons and we were using our ReFOLD3 server method for refinement. The table is sorted by GDTTS_cons score. Data are from <https://predictioncenter.org/caspcommons/models_consensus2.cgi>.

| Ranking | Model Name | Predictor | LDDT_cons | GDTTS_cons |
| --- | --- | --- | --- | --- |
| 1 | MULTICOM | 0.285 | 0.14 |  |
| 2 | C1901TS044_1 | FEIGLAB | 0.287 | 0.14 |
| **3** | **C1901TS213_1** | **McGuffin (ReFOLD3)** | **0.291** | **0.139** |
| **4** | **C1901TS213_3** | **McGuffin (ReFOLD3)** | **0.29** | **0.139** |
| **5** | **C1901TS213_5** | **McGuffin (ReFOLD3)** | **0.29** | **0.139** |
| **6** | **C1901TS213_2** | **McGuffin (ReFOLD3)** | **0.29** | **0.139** |
| **7** | **C1901TS213_4** | **McGuffin (ReFOLD3)** | **0.289** | **0.139** |
| 8 | C1901TS228_1 | DellaCorteLab | 0.288 | 0.136 |
| 9 | C1901TS273_1 | Takeda-Shitaka-Lab | 0.284 | 0.128 |
| 10 | C1901TS215_5 | PerezLab_Gators | 0.252 | 0.111 |
| 11 | C1901TS215_3 | PerezLab_Gators | 0.251 | 0.111 |
| 12 | C1901TS215_4 | PerezLab_Gators | 0.247 | 0.111 |
| 13 | C1901TS215_1 | PerezLab_Gators | 0.248 | 0.11 |
| 14 | C1901TS215_2 | PerezLab_Gators | 0.249 | 0.11 |
| 15 | C1901TS438_5 | Destini | 0.248 | 0.108 |
| 16 | C1901TS438_2 | Destini | 0.241 | 0.107 |
| 17 | C1901TS438_1 | Destini | 0.246 | 0.107 |
| 18 | C1901TS438_4 | Destini | 0.245 | 0.107 |
| 19 | C1901TS152_2 | MULTICOM | 0.276 | 0.105 |
| 20 | C1901TS413_3 | TFold-server | 0.273 | 0.105 |

Supplementary Table S4. Official CASP Commons results for C1902 according to the consensus GDT-TS and LDDT scores for the top 20 models. The table is sorted by GDTTS_cons score. Data are

from <https://predictioncenter.org/caspcommons/models_consensus2.cgi>.

| Ranking | Model Name | Predictor | LDDT_cons | GDTTS_cons |
| --- | --- | --- | --- | --- |
| 1 | C1902TS413_4 | TFold-server | 0.344 | 0.187 |
| **2** | **C1902TS213_1** | **McGuffin (ReFOLD3)** | **0.347** | **0.182** |
| **3** | **C1902TS213_2** | **McGuffin (ReFOLD3)** | **0.348** | **0.182** |
| **4** | **C1902TS213_4** | **McGuffin (ReFOLD3)** | **0.347** | **0.182** |
| **5** | **C1902TS213_3** | **McGuffin (ReFOLD3)** | **0.348** | **0.181** |
| **6** | **C1902TS213_5** | **McGuffin (ReFOLD3)** | **0.346** | **0.181** |
| 7 | C1902TS152_4 | MULTICOM | 0.346 | 0.18 |
| 8 | C1902TS152_2 | MULTICOM | 0.346 | 0.173 |
| 9 | C1902TS413_1 | TFold-server | 0.348 | 0.173 |
| 10 | C1902TS273_2 | Takeda-Shitaka-Lab | 0.33 | 0.169 |
| 11 | C1902TS413_3 | TFold-server | 0.331 | 0.168 |
| 12 | C1902TS152_3 | MULTICOM | 0.332 | 0.168 |
| 13 | C1902TS438_3 | Destini | 0.369 | 0.168 |
| 14 | C1902TS438_4 | Destini | 0.352 | 0.167 |
| 15 | C1902TS413_2 | TFold-server | 0.336 | 0.167 |
| 16 | C1902TS152_5 | MULTICOM | 0.352 | 0.167 |
| 17 | C1902TS413_5 | TFold-server | 0.345 | 0.167 |
| 18 | C1902TS438_1 | Destini | 0.36 | 0.167 |
| 19 | C1902TS438_2 | Destini | 0.37 | 0.167 |
| 20 | C1902TS438_5 | Destini | 0.362 | 0.16 |

Supplementary Table S5. Official CASP Commons results for C1903 according to the consensus GDT-TS and LDDT scores for the top 20 models. The table is sorted by GDTTS_cons score. Data are from <https://predictioncenter.org/caspcommons/models_consensus2.cgi>.

| Ranking | Model Name | Predictor | LDDT_cons | GDTTS_cons |
| --- | --- | --- | --- | --- |
| 1 | C1903TS044_1 | FEIGLAB | 0.345 | 0.212 |
| 2 | C1903TS301_1 | FEIGLAB-S | 0.345 | 0.212 |
| **3** | **C1903TS213_5** | **McGuffin (ReFOLD3)** | **0.348** | **0.21** |
| **4** | **C1903TS213_4** | **McGuffin (ReFOLD3)** | **0.349** | **0.21** |
| **5** | **C1903TS213_2** | **McGuffin (ReFOLD3)** | **0.347** | **0.21** |
| **6** | **C1903TS213_1** | **McGuffin (ReFOLD3)** | **0.347** | **0.21** |
| 7 | C1903TS438_1 | Destini | 0.353 | 0.209 |
| 8 | C1903TS438_4 | Destini | 0.352 | 0.209 |
| 9 | C1903TS438_5 | Destini | 0.353 | 0.209 |
| 10 | C1903TS438_3 | Destini | 0.35 | 0.209 |
| **11** | **C1903TS213_3** | **McGuffin (ReFOLD3)** | **0.347** | **0.209** |
| 12 | C1903TS438_2 | Destini | 0.351 | 0.207 |
| 13 | C1903TS228_1 | DellaCorteLab | 0.347 | 0.207 |
| 14 | C1903TS152_2 | MULTICOM | 0.363 | 0.203 |
| 15 | C1903TS413_5 | TFold-server | 0.356 | 0.203 |
| 16 | C1903TS152_1 | MULTICOM | 0.358 | 0.203 |
| 17 | C1903TS247_1 | AWSEM-Suite-Commons | 0.339 | 0.201 |
| 18 | C1903TS247_2 | AWSEM-Suite-Commons | 0.339 | 0.2 |
| 19 | C1903TS413_3 | TFold-server | 0.356 | 0.2 |
| 20 | C1903TS152_3 | MULTICOM | 0.342 | 0.199 |

Supplementary Table S6. Official CASP Commons results for C1904 according to the consensus GDT-TS and LDDT scores for the top 20 models. The table is sorted by GDTTS_cons score. Data are from <https://predictioncenter.org/caspcommons/models_consensus2.cgi>.

| Ranking | Model Name | Predictor | LDDT_cons | GDTTS_cons |
| --- | --- | --- | --- | --- |
| 1 | C1904TS401_1 | FEIGLAB-R | 0.347 | 0.183 |
| 2 | C1904TS273_1 | Takeda-Shitaka-Lab | 0.31 | 0.151 |
| 3 | C1904TS044_1 | FEIGLAB | 0.312 | 0.151 |
| **4** | **C1904TS213_2** | **McGuffin (ReFOLD3)** | **0.314** | **0.15** |
| **5** | **C1904TS213_4** | **McGuffin (ReFOLD3)** | **0.313** | **0.15** |
| **6** | **C1904TS213_5** | **McGuffin (ReFOLD3)** | **0.314** | **0.15** |
| 7 | C1904TS152_2 | MULTICOM | 0.309 | 0.15 |
| **8** | **C1904TS213_3** | **McGuffin (ReFOLD3)** | **0.313** | **0.15** |
| **9** | **C1904TS213_1** | **McGuffin (ReFOLD3)** | **0.314** | **0.149** |
| 10 | C1904TS228_1 | DellaCorteLab | 0.312 | 0.146 |
| 11 | C1904TS215_5 | PerezLab_Gators | 0.271 | 0.122 |
| 12 | C1904TS215_2 | PerezLab_Gators | 0.266 | 0.121 |
| 13 | C1904TS215_4 | PerezLab_Gators | 0.264 | 0.12 |
| 14 | C1904TS413_5 | TFold-server | 0.298 | 0.119 |
| 15 | C1904TS152_3 | MULTICOM | 0.298 | 0.119 |
| 16 | C1904TS215_3 | PerezLab_Gators | 0.265 | 0.117 |
| 17 | C1904TS438_5 | Destini | 0.301 | 0.117 |
| 18 | C1904TS215_1 | PerezLab_Gators | 0.264 | 0.117 |
| 19 | C1904TS438_1 | Destini | 0.303 | 0.117 |
| 20 | C1904TS438_3 | Destini | 0.303 | 0.117 |

Supplementary Table S7. Official CASP Commons results for C1905 according to the consensus GDT-TS and LDDT scores for the top 20 models. The table is sorted by GDTTS_cons score. Data are from <https://predictioncenter.org/caspcommons/models_consensus2.cgi>.

| Ranking | Model Name | Predictor | LDDT_cons | GDTTS_cons |
| --- | --- | --- | --- | --- |
| 1 | C1905TS413_1 | TFold-server | 0.275 | 0.201 |
| **2** | **C1905TS213_4** | **McGuffin (ReFOLD3)** | **0.285** | **0.2** |
| **3** | **C1905TS213_5** | **McGuffin (ReFOLD3)** | **0.287** | **0.2** |
| **4** | **C1905TS213_3** | **McGuffin (ReFOLD3)** | **0.287** | **0.2** |
| **5** | **C1905TS213_2** | **McGuffin (ReFOLD3)** | **0.286** | **0.199** |
| **6** | **C1905TS213_1** | **McGuffin (ReFOLD3)** | **0.287** | **0.199** |
| 7 | C1905TS401_1 | FEIGLAB-R | 0.298 | 0.198 |
| 8 | C1905TS413_3 | TFold-server | 0.274 | 0.196 |
| 9 | C1905TS152_1 | MULTICOM | 0.266 | 0.19 |
| 10 | C1905TS413_5 | TFold-server | 0.264 | 0.19 |
| 11 | C1905TS152_2 | MULTICOM | 0.259 | 0.188 |
| 12 | C1905TS413_4 | TFold-server | 0.257 | 0.188 |
| 13 | C1905TS413_2 | TFold-server | 0.267 | 0.187 |
| 14 | C1905TS438_5 | Destini | 0.307 | 0.178 |
| 15 | C1905TS438_3 | Destini | 0.305 | 0.178 |
| 16 | C1905TS301_1 | FEIGLAB-S | 0.311 | 0.177 |
| 17 | C1905TS044_1 | FEIGLAB | 0.311 | 0.177 |
| 18 | C1905TS152_3 | MULTICOM | 0.279 | 0.176 |
| 19 | C1905TS196_1 | ntsu | 0.283 | 0.175 |
| 20 | C1905TS102_2 | D-Haven | 0.283 | 0.175 |

Supplementary Table S8. Official CASP Commons results for C1906 according to the consensus GDT-TS and LDDT scores for the top 20 models. The table is sorted by GDTTS_cons score. Data are from <https://predictioncenter.org/caspcommons/models_consensus2.cgi>.

| Ranking | Model Name | Predictor | LDDT_cons | GDTTS_cons |
| --- | --- | --- | --- | --- |
| 1 | C1906TS301_1 | FEIGLAB-S | 0.438 | 0.327 |
| 2 | C1906TS044_1 | FEIGLAB | 0.438 | 0.327 |
| 3 | C1906TS152_3 | MULTICOM | 0.434 | 0.327 |
| 4 | C1906TS438_3 | Destini | 0.43 | 0.324 |
| **5** | **C1906TS213_4** | **McGuffin (ReFOLD3)** | **0.438** | **0.324** |
| 6 | C1906TS152_2 | MULTICOM | 0.428 | 0.323 |
| **7** | **C1906TS213_3** | **McGuffin (ReFOLD3)** | **0.439** | **0.323** |
| 8 | C1906TS438_2 | Destini | 0.43 | 0.323 |
| **9** | **C1906TS213_1** | **McGuffin (ReFOLD3)** | **0.437** | **0.323** |
| 10 | C1906TS438_1 | Destini | 0.433 | 0.323 |
| **11** | **C1906TS213_2** | **McGuffin (ReFOLD3)** | **0.439** | **0.323** |
| 12 | C1906TS438_5 | Destini | 0.43 | 0.323 |
| 13 | C1906TS152_1 | MULTICOM | 0.427 | 0.322 |
| 14 | C1906TS413_1 | TFold-server | 0.418 | 0.322 |
| 15 | C1906TS413_2 | TFold-server | 0.413 | 0.321 |
| 16 | C1906TS413_3 | TFold-server | 0.424 | 0.321 |
| 17 | C1906TS438_4 | Destini | 0.428 | 0.32 |
| **18** | **C1906TS213_5** | **McGuffin (ReFOLD3)** | **0.438** | **0.32** |
| 19 | C1906TS413_4 | TFold-server | 0.421 | 0.319 |
| 20 | C1906TS299_5 | FALCON-DeepFolder | 0.442 | 0.317 |

Supplementary Table S9. Official CASP Commons results for C1908 according to the consensus GDT-TS and LDDT scores for the top 20 models. The table is sorted by GDTTS_cons score. Data are from <https://predictioncenter.org/caspcommons/models_consensus2.cgi>.

| Ranking | Model Name | Predictor | LDDT_cons | GDTTS_cons |
| --- | --- | --- | --- | --- |
| 1 | C1908TS152_2 | MULTICOM | 0.314 | 0.315 |
| 2 | C1908TS413_3 | TFold-server | 0.313 | 0.314 |
| 3 | C1908TS152_1 | MULTICOM | 0.316 | 0.313 |
| 4 | C1908TS273_2 | Takeda-Shitaka-Lab | 0.312 | 0.311 |
| 5 | C1908TS413_2 | TFold-server | 0.313 | 0.311 |
| 6 | C1908TS413_5 | TFold-server | 0.312 | 0.309 |
| 7 | C1908TS438_1 | Destini | 0.315 | 0.309 |
| **8** | **C1908TS213_5** | **McGuffin (ReFOLD3)** | **0.326** | **0.309** |
| 9 | C1908TS273_3 | Takeda-Shitaka-Lab | 0.311 | 0.309 |
| **10** | **C1908TS213_2** | **McGuffin (ReFOLD3)** | **0.327** | **0.309** |
| **11** | **C1908TS213_4** | **McGuffin (ReFOLD3)** | **0.327** | **0.309** |
| 12 | C1908TS299_5 | FALCON-DeepFolder | 0.309 | 0.308 |
| 13 | C1908TS278_5 | FALCON | 0.309 | 0.308 |
| **14** | **C1908TS213_3** | **McGuffin (ReFOLD3)** | **0.326** | **0.308** |
| 15 | C1908TS438_3 | Destini | 0.314 | 0.308 |
| **16** | **C1908TS213_1** | **McGuffin (ReFOLD3)** | **0.326** | **0.307** |
| 17 | C1908TS413_1 | TFold-server | 0.304 | 0.307 |
| 18 | C1908TS438_2 | Destini | 0.316 | 0.307 |
| 19 | C1908TS438_4 | Destini | 0.312 | 0.306 |
| 20 | C1908TS438_5 | Destini | 0.313 | 0.306 |

Supplementary Table S10. Official CASP Commons results for C1909 according to the consensus GDT-TS and LDDT scores for the top 20 models. The table is sorted by GDTTS_cons score. Data are from <https://predictioncenter.org/caspcommons/models_consensus2.cgi>.

| Ranking | Model Name | Predictor | LDDT_cons | GDTTS_cons |
| --- | --- | --- | --- | --- |
| 1 | C1909TS438_5 | Destini | 0.453 | 0.534 |
| 2 | C1909TS123_1 | IntFOLD6 | 0.439 | 0.533 |
| 3 | C1909TS278_1 | FALCON | 0.447 | 0.533 |
| 4 | C1909TS299_1 | FALCON-DeepFolder | 0.447 | 0.533 |
| **5** | **C1909TS213_1** | **McGuffin (ReFOLD3)** | **0.449** | **0.53** |
| 6 | C1909TS123_3 | IntFOLD6 | 0.441 | 0.53 |
| 7 | C1909TS152_4 | MULTICOM | 0.444 | 0.53 |
| 8 | C1909TS158_2 | FALCON-TBM | 0.438 | 0.529 |
| 9 | C1909TS369_1 | Yang | 0.42 | 0.527 |
| **10** | **C1909TS213_4** | **McGuffin (ReFOLD3)** | **0.45** | **0.526** |
| 11 | C1909TS309_1 | Zhang-TBM | 0.435 | 0.526 |
| **12** | **C1909TS213_5** | **McGuffin (ReFOLD3)** | **0.447** | **0.525** |
| **13** | **C1909TS213_3** | **McGuffin (ReFOLD3)** | **0.448** | **0.525** |
| 14 | C1909TS309_5 | Zhang-TBM | 0.435 | 0.525 |
| 15 | C1909TS369_5 | Yang | 0.429 | 0.525 |
| **16** | **C1909TS213_2** | **McGuffin (ReFOLD3)** | **0.451** | **0.525** |
| 17 | C1909TS369_4 | Yang | 0.416 | 0.524 |
| 18 | C1909TS299_4 | FALCON-DeepFolder | 0.445 | 0.523 |
| 19 | C1909TS152_2 | MULTICOM | 0.444 | 0.523 |
| 20 | C1909TS278_4 | FALCON | 0.445 | 0.523 |

Supplementary Table S11. Official CASP14 results for regular refinement targets according to the CASP assessors’ formula (0.46*RMS_CA + 0.2*SG + 0.17*GDT_HA + 0.15*QCS + 0.02*Molprb-Score) for the top 15 groups. Our group was registered as “McGuffin” in CASP14 and we were using our ReFOLD3 server method for refinement. The table is sorted by SUM Zscore (>-0.0). Data are from <https://www.predictioncenter.org/casp14/>.

| Group  Ranking | GR code | GR name | Domains Count | SUM Zscore (>-2.0) | Rank SUM Zscore (>-2.0) | AVG Zscore (>-2.0) | Rank AVG Zscore (>-2.0) | SUM Zscore (>0.0) | Rank SUM Zscore (>0.0) | AVG Zscore (>0.0) | Rank AVG Zscore (>0.0) |
| --- | --- | --- | --- | --- | --- | --- | --- | --- | --- | --- | --- |
| 1 | 473 | BAKER | 37 | 15.1206 | 9 | 0.4087 | 10 | 25.1719 | 1 | 0.6803 | 1 |
| 2 | 335 | FEIG | 37 | 21.6824 | 2 | 0.586 | 2 | 24.121 | 2 | 0.6519 | 2 |
| 3 | 13 | FEIG-S | 37 | 22.1338 | 1 | 0.5982 | 1 | 23.1279 | 3 | 0.6251 | 3 |
| 4 | 323 | DellaCorteLab | 37 | 18.4814 | 4 | 0.4995 | 4 | 20.2914 | 4 | 0.5484 | 4 |
| - | 999 | ---STARTING-MODEL--- | 37 | 18.7087 | 3 | 0.5056 | 3 | 19.3092 | 5 | 0.5219 | 5 |
| 5 | 149 | Bhattacharya-Server | 37 | 17.5465 | 5 | 0.4742 | 5 | 18.2434 | 6 | 0.4931 | 6 |
| 6 | 253 | Bhattacharya | 37 | 17.2288 | 6 | 0.4656 | 6 | 17.9815 | 7 | 0.486 | 7 |
| 7 | 71 | Kiharalab | 37 | 15.7113 | 7 | 0.4246 | 8 | 17.659 | 8 | 0.4773 | 8 |
| 8 | 403 | BAKER-experimental | 37 | -2.5792 | 17 | -0.0697 | 21 | 17.1339 | 9 | 0.4631 | 9 |
| **9** | **220** | **McGuffin (ReFOLD3)** | **37** | **15.5957** | **8** | **0.4215** | **9** | **16.7944** | **10** | **0.4539** | **11** |
| 10 | 100 | AIR | 37 | 12.2328 | 10 | 0.3306 | 11 | 15.4769 | 11 | 0.4183 | 12 |
| 11 | 70 | Seok-server | 37 | 11.4427 | 13 | 0.3093 | 14 | 14.8744 | 12 | 0.402 | 13 |
| 12 | 236 | DeepMUSICS | 37 | 11.9093 | 11 | 0.3219 | 12 | 14.7498 | 13 | 0.3986 | 14 |
| 13 | 394 | Kiharalab_Refine | 37 | 8.7907 | 15 | 0.2376 | 17 | 14.0749 | 14 | 0.3804 | 16 |
| 14 | 470 | PerillaGroup | 37 | 11.8274 | 12 | 0.3197 | 13 | 13.9195 | 15 | 0.3762 | 17 |
| 15 | 360 | UNRES | 37 | 11.2364 | 14 | 0.3037 | 15 | 13.6851 | 16 | 0.3699 | 19 |

Supplementary Table S12. Official CASP14 results for regular refinement targets according to the GDT_TS score for the top 15 groups. The table is sorted by SUM Zscore (>-0.0). Data are from <https://www.predictioncenter.org/casp14/>.

| Group  Ranking | GR code | GR name | Domains Count | SUM Zscore (>-2.0) | Rank SUM Zscore (>-2.0) | AVG Zscore (>-2.0) | Rank AVG Zscore (>-2.0) | SUM Zscore (>0.0) | Rank SUM Zscore (>0.0) | AVG Zscore (>0.0) | Rank AVG Zscore (>0.0) |
| --- | --- | --- | --- | --- | --- | --- | --- | --- | --- | --- | --- |
| 1 | 335 | FEIG | 37 | 31.3864 | 1 | 0.8483 | 1 | 31.987 | 1 | 0.8645 | 1 |
| 2 | 13 | FEIG-S | 37 | 28.6772 | 2 | 0.7751 | 2 | 29.5724 | 2 | 0.7993 | 2 |
| 3 | 473 | BAKER | 37 | 18.4605 | 5 | 0.4989 | 6 | 25.5647 | 3 | 0.6909 | 3 |
| 4 | 323 | DellaCorteLab | 37 | 20.4567 | 3 | 0.5529 | 3 | 20.6283 | 4 | 0.5575 | 4 |
| 5 | 403 | BAKER-experimental | 37 | 4.2479 | 17 | 0.1148 | 20 | 20.3459 | 5 | 0.5499 | 5 |
| - | 999 | ---STARTING-MODEL--- | 37 | 19.7486 | 4 | 0.5337 | 4 | 20.2042 | 6 | 0.5461 | 6 |
| 6 | 71 | Kiharalab | 37 | 16.562 | 8 | 0.4476 | 9 | 18.5737 | 7 | 0.502 | 8 |
| 7 | 236 | DeepMUSICS | 37 | 17.4147 | 6 | 0.4707 | 7 | 18.1326 | 8 | 0.4901 | 9 |
| 8 | 253 | Bhattacharya | 37 | 16.7986 | 7 | 0.454 | 8 | 17.5687 | 9 | 0.4748 | 10 |
| 9 | 149 | Bhattacharya-Server | 37 | 16.5208 | 9 | 0.4465 | 10 | 17.1631 | 10 | 0.4639 | 11 |
| **10** | **220** | **McGuffin (ReFOLD3)** | **37** | **15.5324** | **10** | **0.4198** | **12** | **16.4612** | **11** | **0.4449** | **12** |
| 12 | 70 | Seok-server | 37 | 13.893 | 11 | 0.3755 | 13 | 15.2839 | 12 | 0.4131 | 14 |
| 13 | 360 | UNRES | 37 | 12.7611 | 12 | 0.3449 | 14 | 14.1534 | 13 | 0.3825 | 15 |
| 14 | 100 | AIR | 37 | 12.6743 | 13 | 0.3425 | 15 | 13.995 | 14 | 0.3782 | 16 |
| 15 | 470 | PerillaGroup | 37 | 10.7411 | 14 | 0.2903 | 16 | 13.9325 | 15 | 0.3766 | 17 |

Supplementary Table S13. The performance of our refinement pipeline for on all regular FM targets according to the GDT-TS, lDDT and Molprobity scores versus the starting model. The 3D models were generated by ReFOLD3, and the best-refined 3D model which was selected by ModFOLD8 was submitted during CASP14. Higher GDT-TS, lDDT and lower Molprobity scores are better. Data are from <https://www.predictioncenter.org/casp14/>.

| CASP model ID | | | MoldFOLD8 | | | GDT-TS | | | lDDT | | | Molprobity | | |
| --- | --- | --- | --- | --- | --- | --- | --- | --- | --- | --- | --- | --- | --- | --- |
| Target ID | Starting Model | Submitted Model | Starting  model | Refined  model | Diff | Starting  model | Refined  model | Diff | Starting  mode; | Refined  model | Diff | Starting  model | Refined  model | Diff |
| T1031 | T1031TS209_5-D1 | T1031TS220_1-D1 | 0.5127 | 0.5394 | 0.0267 | 24.47 | 24.74 | 0.27 | 0.32 | 0.32 | 0 | 1.15 | 2.09 | 0.94 |
| T1033 | T1033TS209_5-D1 | T1033TS220_1-D1 | 0.4969 | 0.5148 | 0.0179 | 46.25 | 45.25 | -1 | 0.51 | 0.5 | -0.01 | 1.35 | 1.84 | 0.49 |
| T1037 | T1037TS337_1-D1 | T1037TS220_1-D1 | 0.4191 | 0.4204 | 0.0013 | 51.3 | 51.3 | 0 | 0.52 | 0.53 | 0.01 | 2.8 | 3.62 | 0.82 |
| T1039 | T1039TS487_4-D1 | T1039TS220_1-D1 | 0.3943 | 0.4064 | 0.0121 | 34.63 | 34.78 | 0.15 | 0.35 | 0.38 | 0.03 | 2.16 | 2.44 | 0.28 |
| T1040 | T1040TS351_3-D1 | T1040TS220_1-D1 | 0.4429 | 0.4573 | 0.0144 | 24.42 | 23.65 | -0.77 | 0.38 | 0.38 | 0 | 0.79 | 1.13 | 0.34 |
| T1041 | T1041TS377_3-D1 | T1041TS220_1-D1 | 0.4288 | 0.4408 | 0.012 | 52.58 | 53 | 0.42 | 0.53 | 0.53 | 0 | 2.84 | 1.87 | -0.97 |
| T1042 | T1042TS226_2-D1 | T1042TS220_1-D1 | 0.3835 | 0.399 | 0.0155 | 54.98 | 54.62 | -0.36 | 0.55 | 0.53 | -0.02 | 1.3 | 1.8 | 0.5 |
| T1043 | T1043TS487_3-D1 | T1043TS220_1-D1 | 0.3495 | 0.3634 | 0.0139 | 17.06 | 17.23 | 0.17 | 0.22 | 0.24 | 0.02 | 1.32 | 2.21 | 0.89 |
| T1047s1 | T1047s1TS075_1-D1 | T1047s1TS220_1-D1 | 0.5158 | 0.5325 | 0.0167 | 32.58 | 32.94 | 0.36 | 0.58 | 0.56 | -0.02 | 2.95 | 2.25 | -0.7 |
| T1049 | T1049TS326_3-D1 | T1049TS220_1-D1 | 0.578 | 0.5842 | 0.0062 | 63.25 | 63.43 | 0.18 | 0.57 | 0.58 | 0.01 | 2.63 | 2.68 | 0.05 |
| T1090 | T1090TS487_1-D1 | T1090TS220_1-D1 | 0.546 | 0.5501 | 0.0041 | 53.17 | 53.17 | 0 | 0.51 | 0.53 | 0.02 | 2.02 | 2.75 | 0.73 |
| T1093 | T1093TS487_3 | T1093TS220_1 | 0.4522 | 0.4562 | 0.004 | 25.44 | 25.4 | -0.04 | 0.36 | 0.39 | 0.03 | 2.02 | 2.17 | 0.15 |
| T1027 | T1027TS487_1-D1 | T1027TS220_1-D1 | 0.4736 | 0.483 | 0.0094 | 36.87 | 37.12 | 0.25 | 0.4 | 0.4 | 0 | 1.25 | 2.73 | 1.48 |
| T1029 | T1029TS487_1-D1 | T1029TS220_1-D1 | 0.6367 | 0.6316 | -0.0051 | 40.8 | 40.8 | 0 | 0.47 | 0.47 | 0 | 1.95 | 1.95 | 0 |
| T1064 | T1064TS140_1-D1 | T1064TS220_1-D1 | 0.4853 | 0.4997 | 0.0144 | 20.65 | 20.65 | 0 | 0.22 | 0.23 | 0.01 | 2.39 | 2.96 | 0.57 |
| T1074 | T1074TS487_5-D1 | T1074TS220_1-D1 | 0.4406 | 0.4605 | 0.0199 | 35.8 | 35.98 | 0.18 | 0.38 | 0.39 | 0.01 | 2.02 | 2.69 | 0.67 |
| T1096 | T1096TS252_1 | T1096TS220_1 | 0.4619 | 0.4831 | 0.0212 | 26.14 | 26.52 | 0.38 | 0.52 | 0.56 | 0.04 | 2.54 | 2.34 | -0.2 |
| Total | | | 8.0178 | 8.2224 | 0.2046 | 640.39 | 640.58 | 0.19 | 7.39 | 7.52 | 0.13 | 33.48 | 39.52 | 6.04 |

Supplementary Table S14. The performance of our refinement pipeline for on all regular TBM targets according to the GDT-TS, lDDT and Molprobity scores versus the starting model. The 3D models were generated by ReFOLD3, and the best-refined 3D model which was selected by ModFOLD8 was submitted during CASP14. Higher GDT-TS, lDDT and lower Molprobity scores are better. Data are from <https://www.predictioncenter.org/casp14>.

| CASP model ID | | | MoldFOLD8 | | | GDT-TS | | | lDDT | | | Molprobity | | |
| --- | --- | --- | --- | --- | --- | --- | --- | --- | --- | --- | --- | --- | --- | --- |
| Target ID | Starting Model | Submitted Model | Starting  model | Refined  model | Diff | Starting  model | Refined  model | Diff | Starting  model | Refined  model | Diff | Starting  model | Refined | Diff |
| T1024 | T1024TS326_2 | T1024TS220_1 | 0.7008 | 0.7025 | 0.0017 | 60.68 | 60.93 | 0.25 | 0.67 | 0.68 | 0.01 | 2.12 | 1.84 | -0.28 |
| T1026 | T1026TS487_4-D1 | T1026TS220_1-D1 | 0.4914 | 0.4981 | 0.0067 | 68.49 | 68.15 | -0.34 | 0.54 | 0.58 | 0.04 | 1.72 | 2.68 | 0.96 |
| T1030 | T1030TS487_1 | T1030TS220_1 | 0.5104 | 0.5153 | 0.0049 | 39.84 | 43.77 | 3.93 | 0.6 | 0.62 | 0.02 | 1.59 | 1.64 | 0.05 |
| T1034 | T1034TS487_1-D1 | T1034TS220_1-D1 | 0.7408 | 0.7423 | 0.0015 | 82.37 | 82.53 | 0.16 | 0.7 | 0.73 | 0.03 | 1.71 | 1.82 | 0.11 |
| T1045s2 | T1045s2TS487_4-D1 | T1045s2TS220_1-D1 | 0.6457 | 0.6507 | 0.005 | 69.28 | 70.03 | 0.75 | 0.63 | 0.65 | 0.02 | 1.57 | 2.04 | 0.47 |
| T1046s2 | T1046s2TS487_4-D1 | T1046s2TS220_1-D1 | 0.5867 | 0.5918 | 0.0051 | 75.53 | 76.06 | 0.53 | 0.57 | 0.63 | 0.06 | 2.59 | 2.67 | 0.08 |
| T1050 | T1050TS487_1 | T1050TS220_1 | 0.7227 | 0.7237 | 0.001 | 55.85 | 55.88 | 0.03 | 0.63 | 0.67 | 0.04 | 1.91 | 2.12 | 0.21 |
| T1054 | T1054TS326_4-D1 | T1054TS220_1-D1 | 0.6271 | 0.628 | 0.0009 | 67.48 | 68.71 | 1.23 | 0.66 | 0.68 | 0.02 | 1.96 | 2.03 | 0.07 |
| T1056 | T1056TS209_3-D1 | T1056TS220_1-D1 | 0.6119 | 0.6229 | 0.011 | 54.44 | 53.25 | -1.19 | 0.48 | 0.46 | -0.02 | 1.47 | 2.7 | 1.23 |
| T1057 | T1057TS351_5-D1 | T1057TS220_1-D1 | 0.7007 | 0.7062 | 0.0055 | 79.57 | 79.47 | -0.1 | 0.68 | 0.68 | 0 | 2.02 | 1.96 | -0.06 |
| T1060s3 | T1060s3TS487_4-D1 | T1060s3TS220_1-D1 | 0.657 | 0.6625 | 0.0055 | 67.89 | 67.28 | -0.61 | 0.61 | 0.64 | 0.03 | 1.83 | 2.48 | 0.65 |
| T1065s1 | T1065s1TS487_1-D1 | T1065s1TS220_1-D1 | 0.6707 | 0.6817 | 0.011 | 88.44 | 88.87 | 0.43 | 0.75 | 0.8 | 0.05 | 1.54 | 1.93 | 0.39 |
| T1065s2 | T1065s2TS277_4-D1 | T1065s2TS220_1-D1 | 0.7145 | 0.7188 | 0.0043 | 90.31 | 91.07 | 0.76 | 0.78 | 0.78 | 0 | 0.52 | 1.56 | 1.04 |
| T1067 | T1067TS351_3-D1 | T1067TS220_1-D1 | 0.5002 | 0.5185 | 0.0183 | 52.83 | 52.49 | -0.34 | 0.51 | 0.49 | -0.02 | 1.61 | 2.63 | 1.02 |
| T1068 | T1068TS183_2-D1 | T1068TS220_1-D1 | 0.5177 | 0.5243 | 0.0066 | 57.26 | 55.17 | -2.09 | 0.53 | 0.51 | -0.02 | 1.19 | 1.82 | 0.63 |
| T1073 | T1073TS140_5-D1 | T1073TS220_1-D1 | 0.6506 | 0.6508 | 0.0002 | 83.47 | 83.47 | 0 | 0.73 | 0.73 | 0 | 2.08 | 1.73 | -0.35 |
| T1076 | T1076TS487_4-D1 | T1076TS220_1-D1 | 0.7285 | 0.7331 | 0.0046 | 87.41 | 87.41 | 0 | 0.72 | 0.78 | 0.06 | 2.04 | 2.16 | 0.12 |
| T1078 | T1078TS351_3-D1 | T1078TS220_1-D1 | 0.6132 | 0.6279 | 0.0147 | 76.74 | 76.55 | -0.19 | 0.7 | 0.69 | -0.01 | 1.25 | 2.46 | 1.21 |
| T1079 | T1079TS487_2-D1 | T1079TS220_1-D1 | 0.7072 | 0.7097 | 0.0025 | 62.03 | 62.36 | 0.33 | 0.64 | 0.68 | 0.04 | 2.06 | 2.19 | 0.13 |
| T1083 | T1083TS364_4-D1 | T1083TS220_1-D1 | 0.6821 | 0.6876 | 0.0055 | 83.7 | 83.15 | -0.55 | 0.71 | 0.71 | 0 | 2.37 | 1.03 | -1.34 |
| T1084 | T1084TS252_2-D1 | T1084TS220_1-D1 | 0.6508 | 0.6555 | 0.0047 | 88.73 | 88.03 | -0.7 | 0.76 | 0.75 | -0.01 | 1.98 | 0.78 | -1.2 |
| T1089 | T1089TS238_4-D1 | T1089TS220_1-D1 | 0.6435 | 0.6494 | 0.0059 | 65.45 | 65.92 | 0.47 | 0.59 | 0.59 | 0 | 1.96 | 2.14 | 0.18 |
| T1091 | T1091TS351_4 | T1091TS220_1 | 0.5052 | 0.513 | 0.0078 | 26.62 | 26.67 | 0.05 | 0.62 | 0.62 | 0 | 1.86 | 2.25 | 0.39 |
| T1092 | T1092TS319_3 | T1092TS220_1 | 0.5231 | 0.535 | 0.0119 | 23.24 | 23.47 | 0.23 | 0.45 | 0.49 | 0.04 | 3.01 | 2.56 | -0.45 |
| T1095 | T1095TS487_2-D1 | T1095TS220_1-D1 | 0.5328 | 0.5404 | 0.0076 | 41.86 | 42.09 | 0.23 | 0.56 | 0.61 | 0.05 | 1.96 | 2.06 | 0.1 |
| T1099 | T1099TS487_4-D1 | T1099TS220_1-D1 | 0.4276 | 0.4408 | 0.0132 | 57.3 | 55.76 | -1.54 | 0.59 | 0.53 | -0.06 | 1.48 | 1.56 | 0.08 |
| T1101 | T1101TS487_4 | T1101TS220_1 | 0.6859 | 0.6874 | 0.0015 | 56.65 | 57.16 | 0.51 | 0.65 | 0.69 | 0.04 | 1.85 | 2.45 | 0.6 |
| Total | | | 16.7488 | 16.9179 | 0.1691 | 1763.46 | 1765.7 | 2.24 | 17.06 | 17.47 | 0.41 | 49.25 | 55.29 | 6.04 |

Supplementary Table S15. The performance of our refinement pipeline for on all regular FM/TBM targets according to the GDT-TS, lDDT and Molprobity scores versus the starting model. The 3D models were generated by ReFOLD3, and the best-refined 3D model which was selected by ModFOLD8 was submitted during CASP14. Higher GDT-TS, lDDT and lower Molprobity scores are better. Data are from <https://www.predictioncenter.org/casp14>.

| CASP model ID | | | MoldFOLD8 | | | GDT-TS | | | lDDT | | | Molprobity | | |
| --- | --- | --- | --- | --- | --- | --- | --- | --- | --- | --- | --- | --- | --- | --- |
| Target ID | Starting Model | Submitted Model | Starting  model | Refined  model | Diff | Starting  model | Refined  model | Diff | Starting  model | Refined  model | Diff | Starting  Model | Refined  model | Diff |
| T1035 | T1035TS351_3-D1 | T1035TS220_1-D1 | 0.5339 | 0.5462 | 0.0123 | 48.28 | 50 | 1.72 | 0.5 | 0.5 | 0 | 1.15 | 1.61 | 0.46 |
| T1038 | T1038TS487_4 | T1038TS220_1 | 0.4848 | 0.4971 | 0.0123 | 26.45 | 26.58 | 0.13 | 0.37 | 0.36 | -0.01 | 1.86 | 2.33 | 0.47 |
| T1046s1 | T1046s1TS487_3-D1 | T1046s1TS220_1-D1 | 0.6743 | 0.6745 | 0.0002 | 74.31 | 75 | 0.69 | 0.6 | 0.6 | 0 | 0.76 | 1.99 | 1.23 |
| T1047s2 | T1047s2TS487_5 | T1047s2TS220_1 | 0.5881 | 0.5903 | 0.0022 | 34.05 | 34.21 | 0.16 | 0.62 | 0.66 | 0.04 | 1.62 | 1.96 | 0.34 |
| T1052 | T1052TS487_4 | T1052TS220_1 | 0.4532 | 0.4657 | 0.0125 | 54.48 | 54.15 | -0.33 | 0.67 | 0.66 | -0.01 | 3.21 | 3.02 | -0.19 |
| T1053 | T1053TS238_4 | T1053TS220_1 | 0.5724 | 0.5805 | 0.0081 | 39.95 | 39.18 | -0.77 | 0.54 | 0.53 | -0.01 | 2.83 | 2.61 | -0.22 |
| T1061 | T1061TS326_4 | T1061TS220_1 | 0.4749 | 0.4762 | 0.0013 | 30.34 | 30.5 | 0.16 | 0.45 | 0.47 | 0.02 | 2.83 | 2.7 | -0.13 |
| T1080 | T1080TS183_1-D1 | T1080TS220_1-D1 | 0.4634 | 0.48 | 0.0166 | 24.81 | 25.19 | 0.38 | 0.41 | 0.41 | 0 | 1.37 | 1.9 | 0.53 |
| T1082 | T1082TS487_5-D1 | T1082TS220_1-D1 | 0.5066 | 0.5423 | 0.0357 | 58.67 | 59.33 | 0.66 | 0.43 | 0.42 | -0.01 | 1.26 | 1.7 | 0.44 |
| T1055 | T1055TS238_5-D1 | T1055TS220_1-D1 | 0.5721 | 0.5802 | 0.0081 | 70.9 | 70.29 | -0.61 | 0.57 | 0.55 | -0.02 | 1.92 | 1.07 | -0.85 |
| T1085 | T1085TS183_3 | T1085TS220_1 | 0.6269 | 0.6343 | 0.0074 | 35.97 | 35.78 | -0.19 | 0.61 | 0.61 | 0 | 1.46 | 1.82 | 0.36 |
| T1086 | T1086TS238_1 | T1086TS220_1 | 0.6753 | 0.6875 | 0.0122 | 41.8 | 42.06 | 0.26 | 0.73 | 0.71 | -0.02 | 1.44 | 1.69 | 0.25 |
| Total | | | 6.6259 | 6.7548 | 0.1289 | 540.01 | 542.27 | 2.26 | 6.5 | 6.48 | -0.02 | 21.71 | 24.4 | 2.69 |

Supplementary Table S16. The performance of our refinement pipeline for all refinement targets according to the GDT-TS, lDDT and Molprobity scores versus the starting model. The 3D models were generated by ReFOLD3, and the best-refined 3D model which was selected by ModFOLD8 was submitted during CASP14. Higher GDT-TS, lDDT and lower Molprobity scores are better. Data are from <https://www.predictioncenter.org/casp14/>.

| CASP model ID | | MoldFOLD8 | | | GDT-TS | | | LDDT | | | Molprobity | | |
| --- | --- | --- | --- | --- | --- | --- | --- | --- | --- | --- | --- | --- | --- |
| Target ID | Submitted model | Starting  Model | Refined  Model | Diff | Starting  model | Refined  Model | Diff | Starting  model | Refined | Diff | Starting | Refined | Diff |
| R1029 | R1029TS220_1 | 0.5745 | 0.5987 | 0.0242 | 46.2 | 45.6 | -0.6 | 0.47 | 0.47 | 0 | 3.96 | 2.62 | -1.34 |
| R1030-D2 | R1030-D2TS220_1 | 0.5176 | 0.5431 | 0.0255 | 59.87 | 60.29 | 0.42 | 0.58 | 0.57 | -0.01 | 0.5 | 1.09 | 0.59 |
| R1031 | R1031TS220_1 | 0.3955 | 0.4264 | 0.0309 | 71.05 | 71.05 | 0 | 0.58 | 0.59 | 0.01 | 0.76 | 2.33 | 1.57 |
| R1033 | R1033TS220_1 | 0.4922 | 0.5131 | 0.0209 | 59 | 57 | -2 | 0.57 | 0.57 | 0 | 1.44 | 2.37 | 0.93 |
| R1034 | R1034TS220_1 | 0.7215 | 0.7337 | 0.0122 | 85.1 | 83.81 | -1.29 | 0.75 | 0.74 | -0.01 | 1.17 | 1.75 | 0.58 |
| R1035 | R1035TS220_1 | 0.4535 | 0.4904 | 0.0369 | 86.77 | 86.28 | -0.49 | 0.76 | 0.75 | -0.01 | 0.86 | 1.38 | 0.52 |
| R1038-D2 | R1038-D2TS220_1 | 0.5475 | 0.5549 | 0.0074 | 74.67 | 74.34 | -0.33 | 0.6 | 0.63 | 0.03 | 3.03 | 2.7 | -0.33 |
| R1039 | R1039TS220_1 | 0.3521 | 0.3777 | 0.0256 | 56.68 | 57.92 | 1.24 | 0.51 | 0.49 | -0.02 | 1.63 | 1.47 | -0.16 |
| R1040v1 | R1040v1TS220_1 | 0.4021 | 0.4026 | 0.0005 | 71.73 | 71.73 | 0 | 0.74 | 0.7 | -0.04 | 0.5 | 0.99 | 0.49 |
| R1040v2 | R1040v2TS220_1 | 0.4106 | 0.4254 | 0.0148 | 47.31 | 46.73 | -0.58 | 0.54 | 0.53 | -0.01 | 2.5 | 2.56 | 0.06 |
| R1041v1 | R1041v1TS220_1 | 0.4408 | 0.4604 | 0.0196 | 87.81 | 85.95 | -1.86 | 0.81 | 0.78 | -0.03 | 1.44 | 1.55 | 0.11 |
| R1041v2 | R1041v2TS220_1 | 0.4063 | 0.4381 | 0.0318 | 63.84 | 62.81 | -1.03 | 0.61 | 0.61 | 0 | 1.19 | 2.46 | 1.27 |
| R1042v1 | R1042v1TS220_1 | 0.3632 | 0.3806 | 0.0174 | 56.43 | 55.98 | -0.45 | 0.59 | 0.56 | -0.03 | 1.15 | 1.44 | 0.29 |
| R1042v2 | R1042v2TS220_1 | 0.3729 | 0.3845 | 0.0116 | 84.51 | 80.71 | -3.8 | 0.83 | 0.78 | -0.05 | 1.31 | 1.34 | 0.03 |
| R1043v1 | R1043v1TS220_1 | 0.2933 | 0.3022 | 0.0089 | 64.02 | 63.01 | -1.01 | 0.6 | 0.59 | -0.01 | 1.18 | 1.6 | 0.42 |
| R1043v2 | R1043v2TS220_1 | 0.3111 | 0.3158 | 0.0047 | 83.45 | 81.25 | -2.2 | 0.75 | 0.73 | -0.02 | 1.2 | 0.92 | -0.28 |
| R1045s2 | R1045s2TS220_1 | 0.6355 | 0.6549 | 0.0194 | 78.31 | 78.01 | -0.3 | 0.68 | 0.69 | 0.01 | 0.89 | 1.82 | 0.93 |
| R1049 | R1049TS220_1 | 0.5478 | 0.5659 | 0.0181 | 71.27 | 71.64 | 0.37 | 0.63 | 0.63 | 0 | 1.2 | 1.93 | 0.73 |
| R1052-D2 | R1052-D2TS220_1 | 0.4779 | 0.4886 | 0.0107 | 78.05 | 77.93 | -0.12 | 0.63 | 0.63 | 0 | 1.32 | 2.29 | 0.97 |
| R1053v1 | R1053v1TS220_1 | 0.5849 | 0.61 | 0.0251 | 72.66 | 71.64 | -1.02 | 0.66 | 0.64 | -0.02 | 0.83 | 1.44 | 0.61 |
| R1053v2 | R1053v2TS220_1 | 0.5884 | 0.6092 | 0.0208 | 92.98 | 91.67 | -1.31 | 0.83 | 0.8 | -0.03 | 0.5 | 1.23 | 0.73 |
| R1055 | R1055TS220_1 | 0.5908 | 0.6187 | 0.0279 | 78.48 | 79.3 | 0.82 | 0.61 | 0.63 | 0.02 | 0.6 | 2.04 | 1.44 |
| R1056 | R1056TS220_1 | 0.6065 | 0.6254 | 0.0189 | 65.24 | 61.39 | -3.85 | 0.55 | 0.51 | -0.04 | 2.29 | 2.49 | 0.2 |
| R1057 | R1057TS220_1 | 0.6785 | 0.6944 | 0.0159 | 84.2 | 85 | 0.8 | 0.73 | 0.72 | -0.01 | 1.14 | 2.4 | 1.26 |
| R1061-D3 | R1061-D3TS220_1 | 0.6304 | 0.6295 | -0.0009 | 78.64 | 78.64 | 0 | 0.61 | 0.61 | 0 | 2.96 | 2.96 | 0 |
| R1065s1 | R1065s1TS220_1 | 0.683 | 0.6974 | 0.0144 | 91.18 | 89.08 | -2.1 | 0.81 | 0.79 | -0.02 | 0.82 | 1.18 | 0.36 |
| R1065s2 | R1065s2TS220_1 | 0.7095 | 0.7234 | 0.0139 | 90.56 | 88.78 | -1.78 | 0.77 | 0.76 | -0.01 | 1.14 | 1.44 | 0.3 |
| R1067v1 | R1067v1TS220_1 | 0.5443 | 0.5551 | 0.0108 | 62.67 | 62.33 | -0.34 | 0.55 | 0.55 | 0 | 1.1 | 1.75 | 0.65 |
| R1067v2 | R1067v2TS220_1 | 0.5537 | 0.5657 | 0.012 | 89.59 | 89.25 | -0.34 | 0.87 | 0.86 | -0.01 | 0.89 | 1.78 | 0.89 |
| R1068 | R1068TS220_1 | 0.503 | 0.53 | 0.027 | 59.92 | 56.15 | -3.77 | 0.55 | 0.52 | -0.03 | 1.33 | 1.43 | 0.1 |
| R1074v1 | R1074v1TS220_1 | 0.5066 | 0.5303 | 0.0237 | 89.77 | 89.77 | 0 | 0.84 | 0.83 | -0.01 | 1.05 | 2.33 | 1.28 |
| R1074v2 | R1074v2TS220_1 | 0.4928 | 0.4867 | -0.0061 | 56.44 | 56.44 | 0 | 0.5 | 0.5 | 0 | 2.04 | 2.04 | 0 |
| R1078 | R1078TS220_1 | 0.6143 | 0.6423 | 0.028 | 80.43 | 80.62 | 0.19 | 0.69 | 0.69 | 0 | 1.25 | 2.75 | 1.5 |
| R1082 | R1082TS220_1 | 0.5039 | 0.512 | 0.0081 | 72.67 | 71.33 | -1.34 | 0.58 | 0.56 | -0.02 | 0.78 | 0.77 | -0.01 |
| R1085-D1 | R1085-D1TS220_1 | 0.571 | 0.5857 | 0.0147 | 62.5 | 63.44 | 0.94 | 0.65 | 0.68 | 0.03 | 2.25 | 1.95 | -0.3 |
| R1090 | R1090TS220_1 | 0.5252 | 0.5449 | 0.0197 | 65.61 | 67.06 | 1.45 | 0.58 | 0.59 | 0.01 | 1.48 | 2.52 | 1.04 |
| R1091-D2 | R1091-D2TS220_1 | 0.5335 | 0.5475 | 0.014 | 79.21 | 81.78 | 2.57 | 0.69 | 0.72 | 0.03 | 1.58 | 2.25 | 0.67 |
| Total | | 19.1362 | 19.7652 | 0.629 | 2698.82 | 2675.71 | -23.11 | 24.3 | 24 | -0.3 | 51.26 | 69.36 | 18.1 |

Supplementary Table S17. The performance of our refinement pipeline for on all refinement FM targets according to the GDT-TS, lDDT and Molprobity scores versus the starting model. The 3D models were generated by ReFOLD3, and the best-refined 3D model which was selected by ModFOLD8 was submitted during CASP14. Higher GDT-TS, lDDT and lower Molprobity scores are better. Data are from <https://www.predictioncenter.org/casp14>.

| CASP model ID | | MoldFOLD8 | | | GDT-TS | | | LDDT | | | Molprobity | | |
| --- | --- | --- | --- | --- | --- | --- | --- | --- | --- | --- | --- | --- | --- |
| Target ID | Submitted model | Starting  Model | Refined  Model | Diff | Starting  Model | Refined  Model | Diff | Starting  Model | Refined  Model | Diff | Starting  Model | Refined  Model | Diff |
| R1029 | R1029TS220_1 | 0.5745 | 0.5987 | 0.0242 | 46.2 | 45.6 | -0.6 | 0.47 | 0.47 | 0 | 3.96 | 2.62 | -1.34 |
| R1031 | R1031TS220_1 | 0.3955 | 0.4264 | 0.0309 | 71.05 | 71.05 | 0 | 0.58 | 0.59 | 0.01 | 0.76 | 2.33 | 1.57 |
| [R1033](https://predictioncenter.org/casp14/results.cgi?target=R1033&model=R1033TS220_1&view=prediction) | [R1033TS220_1](https://predictioncenter.org/casp14/results.cgi?target=R1033&model=R1033TS220_1&view=prediction) | 0.4922 | 0.5131 | 0.0209 | 59 | 57 | -2 | 0.57 | 0.57 | 0 | 1.44 | 2.37 | 0.93 |
| [R1039](https://predictioncenter.org/casp14/results.cgi?target=R1039&model=R1039TS220_1&view=prediction) | R1039TS220_1 | 0.3521 | 0.3777 | 0.0256 | 56.68 | 57.92 | 1.24 | 0.51 | 0.49 | -0.02 | 1.63 | 1.47 | -0.16 |
| [R1040v1](https://predictioncenter.org/casp14/results.cgi?target=R1040v1&model=R1040v1TS220_1&view=prediction) | R1040v1TS220_1 | 0.4021 | 0.4026 | 0.0005 | 71.73 | 71.73 | 0 | 0.74 | 0.7 | -0.04 | 0.5 | 0.99 | 0.49 |
| [R1040v2](https://predictioncenter.org/casp14/results.cgi?target=R1040v2&model=R1040v2TS220_1&view=prediction) | [R1040v2TS220_1](https://predictioncenter.org/casp14/results.cgi?target=R1040v2&model=R1040v2TS220_1&view=prediction) | 0.4106 | 0.4254 | 0.0148 | 47.31 | 46.73 | -0.58 | 0.54 | 0.53 | -0.01 | 2.5 | 2.56 | 0.06 |
| [R1041v1](https://predictioncenter.org/casp14/results.cgi?target=R1041v1&model=R1041v1TS220_1&view=prediction) | R1041v1TS220_1 | 0.4408 | 0.4604 | 0.0196 | 87.81 | 85.95 | -1.86 | 0.81 | 0.78 | -0.03 | 1.44 | 1.55 | 0.11 |
| [R1041v2](https://predictioncenter.org/casp14/results.cgi?target=R1041v2&model=R1041v2TS220_1&view=prediction) | R1041v2TS220_1 | 0.4063 | 0.4381 | 0.0318 | 63.84 | 62.81 | -1.03 | 0.61 | 0.61 | 0 | 1.19 | 2.46 | 1.27 |
| [R1042v1](https://predictioncenter.org/casp14/results.cgi?target=R1042v1&model=R1042v1TS220_1&view=prediction) | R1042v1TS220_1 | 0.3632 | 0.3806 | 0.0174 | 56.43 | 55.98 | -0.45 | 0.59 | 0.56 | -0.03 | 1.15 | 1.44 | 0.29 |
| [R1042v2](https://predictioncenter.org/casp14/results.cgi?target=R1042v2&model=R1042v2TS220_1&view=prediction) | [R1042v2TS220_1](https://predictioncenter.org/casp14/results.cgi?target=R1042v2&model=R1042v2TS220_1&view=prediction) | 0.3729 | 0.3845 | 0.0116 | 84.51 | 80.71 | -3.8 | 0.83 | 0.78 | -0.05 | 1.31 | 1.34 | 0.03 |
| R1043v1 | [R1043v1TS220_1](https://predictioncenter.org/casp14/results.cgi?target=R1043v1&model=R1043v1TS220_1&view=prediction) | 0.2933 | 0.3022 | 0.0089 | 64.02 | 63.01 | -1.01 | 0.6 | 0.59 | -0.01 | 1.18 | 1.6 | 0.42 |
| [R1043v2](https://predictioncenter.org/casp14/results.cgi?target=R1043v2&model=R1043v2TS220_1&view=prediction) | [R1043v2TS220_1](https://predictioncenter.org/casp14/results.cgi?target=R1043v2&model=R1043v2TS220_1&view=prediction) | 0.3111 | 0.3158 | 0.0047 | 83.45 | 81.25 | -2.2 | 0.75 | 0.73 | -0.02 | 1.2 | 0.92 | -0.28 |
| [R1049](https://predictioncenter.org/casp14/results.cgi?target=R1049&model=R1049TS220_1&view=prediction) | [R1049TS220_1](https://predictioncenter.org/casp14/results.cgi?target=R1049&model=R1049TS220_1&view=prediction) | 0.5478 | 0.5659 | 0.0181 | 71.27 | 71.64 | 0.37 | 0.63 | 0.63 | 0 | 1.2 | 1.93 | 0.73 |
| [R1074v1](https://predictioncenter.org/casp14/results.cgi?target=R1074v1&model=R1074v1TS220_1&view=prediction) | [R1074v1TS220_1](https://predictioncenter.org/casp14/results.cgi?target=R1074v1&model=R1074v1TS220_1&view=prediction) | 0.5066 | 0.5303 | 0.0237 | 89.77 | 89.77 | 0 | 0.84 | 0.83 | -0.01 | 1.05 | 2.33 | 1.28 |
| [R1074v2](https://predictioncenter.org/casp14/results.cgi?target=R1074v2&model=R1074v2TS220_1&view=prediction) | [R1074v2TS220_1](https://predictioncenter.org/casp14/results.cgi?target=R1074v2&model=R1074v2TS220_1&view=prediction) | 0.4928 | 0.4867 | -0.0061 | 56.44 | 56.44 | 0 | 0.5 | 0.5 | 0 | 2.04 | 2.04 | 0 |
| [R1090](https://predictioncenter.org/casp14/results.cgi?target=R1090&model=R1090TS220_1&view=prediction) | R1090TS220_1 | 0.5252 | 0.5449 | 0.0197 | 65.61 | 67.06 | 1.45 | 0.58 | 0.59 | 0.01 | 1.48 | 2.52 | 1.04 |
| Total | | 6.887 | 7.1533 | 0.2663 | 1075.12 | 1064.65 | -10.47 | 10.15 | 9.95 | -0.2 | 24.03 | 30.47 | 6.44 |

Supplementary Table S18. The performance of our refinement pipeline for on all refinement TBM targets according to the GDT-TS, lDDT and Molprobity scores versus the starting model. The 3D models were generated by ReFOLD3, and the best-refined 3D model which was selected by ModFOLD8 was submitted during CASP14. Higher GDT-TS, lDDT and lower Molprobity scores are better. Data are from <https://www.predictioncenter.org/casp14>.

| CASP model ID | | MoldFOLD8 | | | GDT-TS | | | LDDT | | | Molprobity | | |
| --- | --- | --- | --- | --- | --- | --- | --- | --- | --- | --- | --- | --- | --- |
| Target ID | Submitted model | Starting  Model | Refined  Model | Diff | Starting  Model | Refined  Model | Diff | Starting  Model | Refined  Model | Diff | Starting  Model | Refined  Model | Diff |
| [R1034](https://predictioncenter.org/casp14/results.cgi?target=R1034&model=R1034TS220_1&view=prediction) | [R1034TS220_1](https://predictioncenter.org/casp14/results.cgi?target=R1034&model=R1034TS220_1&view=prediction) | 0.7215 | 0.7337 | 0.0122 | 85.1 | 83.81 | -1.29 | 0.75 | 0.74 | -0.01 | 1.17 | 1.75 | 0.58 |
| [R1052-D2](https://predictioncenter.org/casp14/results.cgi?target=R1052-D2&model=R1052-D2TS220_1&view=prediction) | [R1052-D2TS220_1](https://predictioncenter.org/casp14/results.cgi?target=R1052-D2&model=R1052-D2TS220_1&view=prediction) | 0.4779 | 0.4886 | 0.0107 | 78.05 | 77.93 | -0.12 | 0.63 | 0.63 | 0 | 1.32 | 2.29 | 0.97 |
| [R1057](https://predictioncenter.org/casp14/results.cgi?target=R1057&model=R1057TS220_1&view=prediction) | [R1057TS220_1](https://predictioncenter.org/casp14/results.cgi?target=R1057&model=R1057TS220_1&view=prediction) | 0.6785 | 0.6944 | 0.0159 | 84.2 | 85 | 0.8 | 0.73 | 0.72 | -0.01 | 1.14 | 2.4 | 1.26 |
| [R1061-D3](https://predictioncenter.org/casp14/results.cgi?target=R1061-D3&model=R1061-D3TS220_1&view=prediction) | [R1061-D3TS220_1](https://predictioncenter.org/casp14/results.cgi?target=R1061-D3&model=R1061-D3TS220_1&view=prediction) | 0.6304 | 0.6295 | -0.0009 | 78.64 | 78.64 | 0 | 0.61 | 0.61 | 0 | 2.96 | 2.96 | 0 |
| [R1091-D2](https://predictioncenter.org/casp14/results.cgi?target=R1091-D2&model=R1091-D2TS220_1&view=prediction) | [R1091-D2TS220_1](https://predictioncenter.org/casp14/results.cgi?target=R1091-D2&model=R1091-D2TS220_1&view=prediction) | 0.5335 | 0.5475 | 0.014 | 79.21 | 81.78 | 2.57 | 0.69 | 0.72 | 0.03 | 1.58 | 2.25 | 0.67 |
| [R1030-D2](https://predictioncenter.org/casp14/results.cgi?target=R1030-D2&model=R1030-D2TS220_1&view=prediction) | [R1030-D2TS220_1](https://predictioncenter.org/casp14/results.cgi?target=R1030-D2&model=R1030-D2TS220_1&view=prediction) | 0.5176 | 0.5431 | 0.0255 | 59.87 | 60.29 | 0.42 | 0.58 | 0.57 | -0.01 | 0.5 | 1.09 | 0.59 |
| [R1045s2](https://predictioncenter.org/casp14/results.cgi?target=R1045s2&model=R1045s2TS220_1&view=prediction) | [R1045s2TS220_1](https://predictioncenter.org/casp14/results.cgi?target=R1045s2&model=R1045s2TS220_1&view=prediction) | 0.6355 | 0.6549 | 0.0194 | 78.31 | 78.01 | -0.3 | 0.68 | 0.69 | 0.01 | 0.89 | 1.82 | 0.93 |
| [R1056](https://predictioncenter.org/casp14/results.cgi?target=R1056&model=R1056TS220_1&view=prediction) | [R1056TS220_1](https://predictioncenter.org/casp14/results.cgi?target=R1056&model=R1056TS220_1&view=prediction) | 0.6065 | 0.6254 | 0.0189 | 65.24 | 61.39 | -3.85 | 0.55 | 0.51 | -0.04 | 2.29 | 2.49 | 0.2 |
| [R1065s1](https://predictioncenter.org/casp14/results.cgi?target=R1065s1&model=R1065s1TS220_1&view=prediction) | [R1065s1TS220_1](https://predictioncenter.org/casp14/results.cgi?target=R1065s1&model=R1065s1TS220_1&view=prediction) | 0.683 | 0.6974 | 0.0144 | 91.18 | 89.08 | -2.1 | 0.81 | 0.79 | -0.02 | 0.82 | 1.18 | 0.36 |
| [R1067v1](https://predictioncenter.org/casp14/results.cgi?target=R1067v1&model=R1067v1TS220_1&view=prediction) | [R1067v1TS220_1](https://predictioncenter.org/casp14/results.cgi?target=R1067v1&model=R1067v1TS220_1&view=prediction) | 0.5443 | 0.5551 | 0.0108 | 62.67 | 62.33 | -0.34 | 0.55 | 0.55 | 0 | 1.1 | 1.75 | 0.65 |
| [R1067v2](https://predictioncenter.org/casp14/results.cgi?target=R1067v2&model=R1067v2TS220_1&view=prediction) | [R1067v2TS220_1](https://predictioncenter.org/casp14/results.cgi?target=R1067v2&model=R1067v2TS220_1&view=prediction) | 0.5537 | 0.5657 | 0.012 | 89.59 | 89.25 | -0.34 | 0.87 | 0.86 | -0.01 | 0.89 | 1.78 | 0.89 |
| [R1068](https://predictioncenter.org/casp14/results.cgi?target=R1068&model=R1068TS220_1&view=prediction) | [R1068TS220_1](https://predictioncenter.org/casp14/results.cgi?target=R1068&model=R1068TS220_1&view=prediction) | 0.503 | 0.53 | 0.027 | 59.92 | 56.15 | -3.77 | 0.55 | 0.52 | -0.03 | 1.33 | 1.43 | 0.1 |
| [R1078](https://predictioncenter.org/casp14/results.cgi?target=R1078&model=R1078TS220_1&view=prediction) | [R1078TS220_1](https://predictioncenter.org/casp14/results.cgi?target=R1078&model=R1078TS220_1&view=prediction) | 0.6143 | 0.6423 | 0.028 | 80.43 | 80.62 | 0.19 | 0.69 | 0.69 | 0 | 1.25 | 2.75 | 1.5 |
| [R1085-D1](https://predictioncenter.org/casp14/results.cgi?target=R1085-D1&model=R1085-D1TS220_1&view=prediction) | [R1085-D1TS220_1](https://predictioncenter.org/casp14/results.cgi?target=R1085-D1&model=R1085-D1TS220_1&view=prediction) | 0.571 | 0.5857 | 0.0147 | 62.5 | 63.44 | 0.94 | 0.65 | 0.68 | 0.03 | 2.25 | 1.95 | -0.3 |
| Total | | 8.2707 | 8.4933 | 0.2226 | 1054.91 | 1047.72 | -7.19 | 9.34 | 9.28 | -0.06 | 19.49 | 27.89 | 8.4 |

Supplementary Table S19. The performance of our refinement pipeline for on all refinement FM/TBM targets according to the GDT-TS, lDDT and Molprobity scores versus the starting model. The 3D models were generated by ReFOLD3, and the best-refined 3D model which was selected by ModFOLD8 was submitted during CASP14. Higher GDT-TS, lDDT and lower Molprobity scores are better. Data are from <https://www.predictioncenter.org/casp14>.

| CASP model ID | | MoldFOLD8 | | | GDT-TS | | | LDDT | | | Molprobity | | |
| --- | --- | --- | --- | --- | --- | --- | --- | --- | --- | --- | --- | --- | --- |
| Target ID | Submitted model | Starting  Model | Refined  Model | Diff | Starting  Model | Refined  Model | Diff | Starting  Model | Refined  Model | Diff | Starting  Model | Refined  Model | Diff |
| R1035 | R1035TS220_1 | 0.4535 | 0.4904 | 0.0369 | 86.77 | 86.28 | -0.49 | 0.76 | 0.75 | -0.01 | 0.86 | 1.38 | 0.52 |
| R1038-D2 | R1038-D2TS220_1 | 0.5475 | 0.5549 | 0.0074 | 74.67 | 74.34 | -0.33 | 0.6 | 0.63 | 0.03 | 3.03 | 2.7 | -0.33 |
| R1053v1 | R1053v1TS220_1 | 0.5849 | 0.61 | 0.0251 | 72.66 | 71.64 | -1.02 | 0.66 | 0.64 | -0.02 | 0.83 | 1.44 | 0.61 |
| R1053v2 | R1053v2TS220_1 | 0.5884 | 0.6092 | 0.0208 | 92.98 | 91.67 | -1.31 | 0.83 | 0.8 | -0.03 | 0.5 | 1.23 | 0.73 |
| R1055 | R1055TS220_1 | 0.5908 | 0.6187 | 0.0279 | 78.48 | 79.3 | 0.82 | 0.61 | 0.63 | 0.02 | 0.6 | 2.04 | 1.44 |
| R1065s2 | R1065s2TS220_1 | 0.7095 | 0.7234 | 0.0139 | 90.56 | 88.78 | -1.78 | 0.77 | 0.76 | -0.01 | 1.14 | 1.44 | 0.3 |
| R1082 | R1082TS220_1 | 0.5039 | 0.512 | 0.0081 | 72.67 | 71.33 | -1.34 | 0.58 | 0.56 | -0.02 | 0.78 | 0.77 | -0.01 |
| Total | | 3.9785 | 4.1186 | 0.1401 | 568.79 | 563.34 | -5.45 | 4.81 | 4.77 | -0.04 | 7.74 | 11 | 3.26 |

Supplementary Table S20. The performance of our refinement pipeline for on all refinement targets with less than 100 residues according to the Z-score. The 3D models were generated by ReFOLD3, and the best-refined 3D model which was selected by ModFOLD8 was submitted during CASP14. Higher Z-score is better. The table is sorted by Avg Zscore (>-0.0). Data are from <https://www.predictioncenter.org/casp14/>.

| Group  Ranking | GR_code | GR_name | Domain Counts | SumZ(>-2) | RSumZ(>-2) | AvgZ(>-2) | RAvgZ(>-2) | SumZ(>0) | RSumZ(>0) | AvgZ(>0) | RAvgZ(>0) |
| --- | --- | --- | --- | --- | --- | --- | --- | --- | --- | --- | --- |
| 1 | 323 | DellaCorteLab | 4 | 3.0572 | 1 | 0.7643 | 1 | 3.1988 | 1 | 0.7997 | 2 |
| 2 | 335 | FEIG | 4 | 2.6816 | 2 | 0.6704 | 2 | 2.6995 | 2 | 0.6749 | 3 |
| 3 | 13 | FEIG-S | 4 | 2.4088 | 3 | 0.6022 | 3 | 2.4088 | 3 | 0.6022 | 4 |
| 4 | 193 | Seok | 4 | 2.0421 | 4 | 0.5105 | 4 | 2.2498 | 4 | 0.5624 | 5 |
| 5 | 340 | Pharmulator | 4 | 1.389 | 12 | 0.3472 | 13 | 1.9962 | 5 | 0.4991 | 6 |
| 6 | 149 | Bhattacharya-Server | 4 | 1.9876 | 5 | 0.4969 | 5 | 1.9876 | 6 | 0.4969 | 7 |
| 7 | 270 | Beta | 4 | 0.8111 | 18 | 0.2028 | 19 | 1.9854 | 7 | 0.4963 | 8 |
| 8 | 473 | BAKER | 4 | 1.6723 | 10 | 0.4181 | 10 | 1.9846 | 8 | 0.4961 | 9 |
| 9 | 253 | Bhattacharya | 4 | 1.7978 | 6 | 0.4494 | 6 | 1.8067 | 9 | 0.4517 | 10 |
| **10** | **220** | **McGuffin (ReFOLD3)** | **4** | **1.719** | **7** | **0.4298** | **7** | **1.7742** | **10** | **0.4436** | **11** |
| - | 999 | ---STARTING-MODEL--- | 4 | 1.7102 | 8 | 0.4275 | 8 | 1.7332 | 11 | 0.4333 | 12 |
| 11 | 71 | Kiharalab | 4 | 1.6842 | 9 | 0.421 | 9 | 1.6945 | 12 | 0.4236 | 13 |
| 12 | 70 | Seok-server | 4 | 1.1399 | 14 | 0.285 | 15 | 1.6883 | 13 | 0.4221 | 14 |
| 13 | 75 | MULTICOM-CLUSTER | 4 | 1.6365 | 11 | 0.4091 | 11 | 1.6853 | 14 | 0.4213 | 15 |
| 14 | 403 | BAKER-experimental | 4 | -0.7064 | 23 | -0.1766 | 25 | 1.671 | 15 | 0.4178 | 16 |

Supplementary Table S21. The performance of our refinement pipeline for on all refinement targets with between 100 and 150 residues according to the Z-score. The 3D models were generated by ReFOLD3, and the best-refined 3D model which was selected by ModFOLD8 was submitted during CASP14. Higher Z-score is better. The table is sorted by Avg Zscore (>-0.0). Data are from <https://www.predictioncenter.org/casp14/>.

| Group  Ranking | GR_code | GR_name | Domain  Counts | SumZ(>-2) | RSumZ(>-2) | AvgZ(>-2) | RavgZ(>-2) | SumZ(>0) | RsumZ(>0) | AvgZ(>0) | RAvgZ(>0) |
| --- | --- | --- | --- | --- | --- | --- | --- | --- | --- | --- | --- |
| 1 | 335 | FEIG | 16 | 11.0781 | 1 | 0.6924 | 1 | 11.4789 | 1 | 0.7174 | 1 |
| 2 | 13 | FEIG-S | 16 | 9.5001 | 2 | 0.5938 | 2 | 10.2103 | 2 | 0.6381 | 2 |
| 3 | 473 | BAKER | 16 | 4.151 | 13 | 0.2594 | 14 | 10.1193 | 3 | 0.6325 | 3 |
| 4 | 323 | DellaCorteLab | 16 | 9.3924 | 3 | 0.587 | 3 | 10.0627 | 4 | 0.6289 | 4 |
| - | 999 | ---STARTING-MODEL--- | 16 | 7.9942 | 4 | 0.4996 | 4 | 8.3719 | 5 | 0.5232 | 6 |
| 5 | 403 | BAKER-experimental | 16 | 0.1846 | 17 | 0.0115 | 21 | 8.1156 | 6 | 0.5072 | 8 |
| 6 | 253 | Bhattacharya | 16 | 7.2537 | 5 | 0.4534 | 6 | 7.6466 | 7 | 0.4779 | 9 |
| **7** | **220** | **McGuffin (ReFOLD3)** | **16** | **7.0599** | **6** | **0.4412** | **7** | **7.5854** | **8** | **0.4741** | **10** |
| 8 | 71 | Kiharalab | 16 | 5.9247 | 9 | 0.3703 | 10 | 7.5418 | 9 | 0.4714 | 11 |
| 19 | 149 | Bhattacharya-Server | 16 | 7.0312 | 7 | 0.4394 | 8 | 7.4139 | 10 | 0.4634 | 12 |
| 10 | 394 | Kiharalab_Refine | 16 | 4.9979 | 12 | 0.3124 | 13 | 7.2571 | 11 | 0.4536 | 13 |
| 11 | 100 | AIR | 16 | 5.6995 | 11 | 0.3562 | 12 | 6.9346 | 12 | 0.4334 | 14 |
| 12 | 70 | Seok-server | 16 | 6.1008 | 8 | 0.3813 | 9 | 6.8527 | 13 | 0.4283 | 15 |
| 13 | 470 | PerillaGroup | 16 | 5.6999 | 10 | 0.3562 | 11 | 6.7877 | 14 | 0.4242 | 16 |
| 14 | 294 | JLU_Comp_Struct_Bio | 10 | -10.2766 | 26 | 0.1723 | 18 | 6.1376 | 15 | 0.6138 | 5 |

Supplementary Table S22. The performance of our refinement pipeline for on all refinement targets with between 150 and 200 residues according to the Z-score. The 3D models were generated by ReFOLD3, and the best-refined 3D model which was selected by ModFOLD8 was submitted during CASP14. Higher Z-score is better. The table is sorted by Avg Zscore (>-0.0). Data are from <https://www.predictioncenter.org/casp14/>.

| Group  Ranking | GR_code | GR name | Domains Count | SumZ(>-2) | RSumZ(>-2) | AvgZ(>-2) | RAvgZ(>-2) | SumZ(>0) | RSumZ(>0) | AvgZ(>0) | RAvgZ(>0) |
| --- | --- | --- | --- | --- | --- | --- | --- | --- | --- | --- | --- |
| 1 | 473 | BAKER | 9 | 5.3219 | 3 | 0.5913 | 3 | 7.4432 | 1 | 0.827 | 1 |
| 2 | 335 | FEIG | 9 | 6.5189 | 1 | 0.7243 | 1 | 6.6083 | 2 | 0.7343 | 2 |
| 3 | 13 | FEIG-S | 9 | 6.1104 | 2 | 0.6789 | 2 | 6.2116 | 3 | 0.6902 | 3 |
| 4 | 403 | BAKER-experimental | 9 | 1.5792 | 16 | 0.1755 | 19 | 5.6636 | 4 | 0.6293 | 4 |
| 5 | 294 | JLU_Comp_Struct_Bio | 8 | -0.6293 | 18 | 0.1713 | 20 | 4.8975 | 5 | 0.6122 | 5 |
| - | 999 | ---STARTING-MODEL--- | 9 | 4.3275 | 4 | 0.4808 | 4 | 4.3601 | 6 | 0.4845 | 6 |
| 6 | 323 | DellaCorteLab | 9 | 3.8978 | 8 | 0.4331 | 8 | 4.3239 | 7 | 0.4804 | 7 |
| 7 | 149 | Bhattacharya-Server | 9 | 4.0998 | 5 | 0.4555 | 5 | 4.2284 | 8 | 0.4698 | 8 |
| 8 | 253 | Bhattacharya | 9 | 3.9844 | 6 | 0.4427 | 6 | 4.0974 | 9 | 0.4553 | 9 |
| 9 | 71 | Kiharalab | 9 | 3.9776 | 7 | 0.442 | 7 | 4.0736 | 10 | 0.4526 | 10 |
| 10 | 100 | AIR | 9 | 2.4471 | 11 | 0.2719 | 13 | 3.6185 | 11 | 0.4021 | 11 |
| **11** | **220** | **McGuffin (ReFOLD3)** | **9** | **3.1087** | **9** | **0.3454** | **10** | **3.5146** | **12** | **0.3905** | **13** |
| 12 | 360 | UNRES | 9 | 2.998 | 10 | 0.3331 | 12 | 3.3721 | 13 | 0.3747 | 15 |
| 13 | 70 | Seok-server | 9 | 1.7026 | 14 | 0.1892 | 17 | 3.093 | 14 | 0.3437 | 16 |
| 14 | 193 | Seok | 9 | 1.5839 | 15 | 0.176 | 18 | 2.8998 | 15 | 0.3222 | 17 |

Supplementary Table S23. The performance of our refinement pipeline for on all refinement targets with more than 200 residues according to the Z-score. The 3D models were generated by ReFOLD3, and the best-refined 3D model which was selected by ModFOLD8 was submitted during CASP14. Higher Z-score is better. The table is sorted by Avg Zscore (>-0.0). Data are from <https://www.predictioncenter.org/casp14/>.

| Ranking | GR_code | Gr name | Domains  Count | SumZ(>-2) | RSumZ(>-2) | AvgZ(>-2) | RAvgZ(>-2) | SumZ(>0) | RSumZ(>0) | AvgZ(>0) | RAvgZ(>0) |
| --- | --- | --- | --- | --- | --- | --- | --- | --- | --- | --- | --- |
| 1 | 473 | BAKER | 8 | 4.2822 | 2 | 0.5353 | 2 | 5.6568 | 1 | 0.7071 | 1 |
| - | 999 | ---STARTING-MODEL--- | 8 | 4.6539 | 1 | 0.5817 | 1 | 4.8746 | 2 | 0.6093 | 3 |
| 2 | 3 | SHORTLE | 8 | -4.9616 | 25 | -0.6202 | 27 | 4.6797 | 3 | 0.585 | 4 |
| 3 | 253 | Bhattacharya | 8 | 4.1728 | 3 | 0.5216 | 4 | 4.4608 | 4 | 0.5576 | 5 |
| 4 | 149 | Bhattacharya-Server | 8 | 4.1091 | 4 | 0.5136 | 5 | 4.3743 | 5 | 0.5468 | 6 |
| **5** | **220** | **McGuffin (ReFOLD3)** | **8** | **3.966** | **5** | **0.4958** | **6** | **4.2217** | **6** | **0.5277** | **8** |
| 6 | 71 | Kiharalab | 8 | 3.8636 | 6 | 0.4829 | 7 | 4.0559 | 7 | 0.507 | 9 |
| 7 | 13 | Spider | 8 | 3.482 | 7 | 0.4352 | 8 | 3.7465 | 8 | 0.4683 | 10 |
| 8 | 100 | AIR | 8 | 3.1141 | 8 | 0.3893 | 10 | 3.4632 | 9 | 0.4329 | 12 |
| 9 | 323 | DellaCorteLab | 8 | 2.836 | 9 | 0.3545 | 11 | 3.3409 | 10 | 0.4176 | 13 |
| 10 | 349 | Spider | 7 | 0.7307 | 17 | 0.3901 | 9 | 3.2397 | 11 | 0.4628 | 11 |
| 11 | 236 | DeepMUSICS | 8 | 2.5852 | 11 | 0.3232 | 13 | 3.1906 | 12 | 0.3988 | 14 |
| 12 | 335 | FEIG | 8 | 1.1289 | 16 | 0.1411 | 19 | 3.1822 | 13 | 0.3978 | 15 |
| 13 | 294 | JLU_Comp_Struct_Bio | 5 | -5.1083 | 26 | 0.1783 | 18 | 3.1264 | 14 | 0.6253 | 2 |
| 14 | 360 | UNRES | 8 | 2.5224 | 13 | 0.3153 | 15 | 3.0493 | 15 | 0.3812 | 16 |

Supplementary Table S24. The performance of our refinement pipeline for on all refinement targets with GDT-TS scores between 60 and 70 according to the Z-score. The 3D models were generated by ReFOLD3, and the best-refined 3D model which was selected by ModFOLD8 was submitted during CASP14. Higher Z-score is better. The table is sorted by Avg Zscore (>-0.0). Data are from <https://www.predictioncenter.org/casp14/>.

| Ranking | GR_code | Gr name | Domains Counts | SumZ(>-2) | RSumZ(>-2) | AvgZ(>-2) | RAvgZ(>-2) | SumZ(>0) | RSumZ(>0) | AvgZ(>0) | RAvgZ(>0) |
| --- | --- | --- | --- | --- | --- | --- | --- | --- | --- | --- | --- |
| 1 | 294 | JLU_Comp_Struct_Bio | 5 | -0.1537 | 20 | 0.3693 | 13 | 4.0463 | 1 | 0.8093 | 1 |
| 2 | 13 | FEIG-S | 6 | 3.6419 | 1 | 0.607 | 2 | 3.6877 | 2 | 0.6146 | 3 |
| 3 | 473 | BAKER | 6 | 2.9597 | 7 | 0.4933 | 8 | 3.6449 | 3 | 0.6075 | 4 |
| - | 999 | ---STARTING-MODEL--- | 6 | 3.5767 | 2 | 0.5961 | 3 | 3.5767 | 4 | 0.5961 | 5 |
| 4 | 253 | Bhattacharya | 6 | 3.4679 | 3 | 0.578 | 4 | 3.4828 | 5 | 0.5805 | 6 |
| **5** | **220** | **McGuffin (ReFOLD3)** | **6** | **3.3933** | **4** | **0.5655** | **5** | **3.4355** | **6** | **0.5726** | **7** |
| 6 | 71 | Kiharalab | 6 | 3.3689 | 5 | 0.5615 | 6 | 3.3689 | 7 | 0.5615 | 8 |
| 7 | 149 | Bhattacharya-Server | 6 | 3.2407 | 6 | 0.5401 | 7 | 3.2428 | 8 | 0.5405 | 9 |
| 8 | 335 | FEIG | 6 | 1.8349 | 13 | 0.3058 | 16 | 3.2228 | 9 | 0.5371 | 10 |
| 9 | 349 | Spider | 5 | 1.0671 | 16 | 0.6134 | 1 | 3.1408 | 10 | 0.6282 | 2 |

Supplementary Table S25. The performance of our refinement pipeline for on all refinement targets with GDT-TS scores higher than 70 according to the Z-score. The 3D models were generated by ReFOLD3, and the best-refined 3D model which was selected by ModFOLD8 was submitted during CASP14. Higher Z-score is better. The table is sorted by Avg Zscore (>-0.0). Data are from <https://www.predictioncenter.org/casp14/>.

| Ranking | GR_code | Gr name | Domains Counts | SumZ(>-2) | RSumZ(>-2) | AvgZ(>-2) | RAvgZ(>-2) | SumZ(>0) | RSumZ(>0) | AvgZ(>0) | RAvgZ(>0) |
| --- | --- | --- | --- | --- | --- | --- | --- | --- | --- | --- | --- |
| - | 999 | ---STARTING-MODEL--- | 7 | 5.1556 | 1 | 0.7365 | 1 | 5.1556 | 1 | 0.7365 | 2 |
| 1 | 294 | JLU_Comp_Struct_Bio | 6 | 0.4242 | 18 | 0.404 | 14 | 5.0642 | 2 | 0.844 | 1 |
| 2 | 394 | Kiharalab_Refine | 7 | 4.8129 | 2 | 0.6876 | 2 | 4.815 | 3 | 0.6879 | 3 |
| 3 | 473 | BAKER | 7 | 4.7545 | 3 | 0.6792 | 3 | 4.7545 | 4 | 0.6792 | 4 |
| **4** | **220** | **McGuffin (ReFOLD3)** | **7** | **4.3665** | **4** | **0.6238** | **4** | **4.4019** | **5** | **0.6288** | **5** |
| 5 | 253 | Bhattacharya | 7 | 4.2069 | 5 | 0.601 | 5 | 4.2998 | 6 | 0.6143 | 6 |
| 6 | 71 | Kiharalab | 7 | 4.1925 | 6 | 0.5989 | 6 | 4.2504 | 7 | 0.6072 | 7 |
| 7 | 149 | Bhattacharya-Server | 7 | 4.1865 | 7 | 0.5981 | 7 | 4.2193 | 8 | 0.6028 | 8 |
| 8 | 470 | PerillaGroup | 7 | 3.7629 | 8 | 0.5376 | 9 | 3.7629 | 9 | 0.5376 | 10 |
| 9 | 323 | DellaCorteLab | 7 | 3.4378 | 10 | 0.4911 | 11 | 3.6778 | 10 | 0.5254 | 11 |
